# Surveillance for Violent Deaths — National Violent Death Reporting System, 50 States, the District of Columbia, and Puerto Rico, 2022

**DOI:** 10.15585/mmwr.ss7405a1

**Published:** 2025-06-12

**Authors:** Kaitlin Forsberg, Kameron J. Sheats, Janet M. Blair, Brenda L. Nguyen, Esther Amoakohene, Carter J. Betz, Bridget H. Lyons

**Affiliations:** ^1^Division of Violence Prevention, National Center for Injury Prevention and Control, CDC; ^2^PCI Government Services

## Abstract

**Problem/Condition:**

In 2022, approximately 24,000 persons died of homicide and approximately 49,000 persons died of suicide in the United States, according to the National Vital Statistics System. This report summarizes data from CDC’s National Violent Death Reporting System (NVDRS) on suicides, homicides, legal intervention deaths, unintentional firearm injury deaths, and deaths of undetermined intent that occurred in the 50 states, the District of Columbia, and Puerto Rico in 2022. Results are reported by sex, age group, race and ethnicity, method of injury, type of location where the injury occurred, circumstances of injury, and other selected characteristics. In contrast to the 2021 NVDRS report, which collected data from a subset of states and included suicide data for persons aged ≥10 years, this report includes data from all 50 states, the District of Columbia, and Puerto Rico, and includes suicide data for all ages.

**Period Covered:**

2022.

**Description of System:**

NVDRS collects data from death certificates, coroner and medical examiner reports, and law enforcement reports. This report includes data collected for violent deaths and suicides that occurred in 2022. Data were collected from all 50 states, the District of Columbia, and Puerto Rico. A total of 47 states had statewide data, three states had data from counties representing a subset of their population (32 California counties, representing 68% of its population; 32 Florida counties, representing 70% of its population; and 13 Texas counties, representing 63% of its population), and the District of Columbia and Puerto Rico had jurisdiction-wide data. NVDRS collates information for each death and links deaths that are related (e.g., multiple homicides, homicide followed by suicide, or multiple suicides) into a single incident.

**Results:**

For 2022, NVDRS collected information on 72,127 fatal incidents involving 74,148 deaths that occurred in all 50 states and the District of Columbia. In addition, data were collected for 727 fatal incidents involving 809 deaths in Puerto Rico, which were analyzed separately. Of the 74,148 deaths that occurred in 50 states and the District of Columbia, the majority (60.6%) were suicides, followed by homicides (30.2%), deaths of undetermined intent (7.1%), legal intervention deaths (1.4%) (i.e., deaths caused by law enforcement and other persons with legal authority to use deadly force acting in the line of duty, excluding legal executions, without denoting the lawfulness or legality of the circumstances surrounding the death), and unintentional firearm injury deaths (<1.0%). Of the 809 deaths that occurred in Puerto Rico, 73.9% were homicides and 23.5% were suicides.

Demographic patterns and circumstances varied by manner of death. In the 50 states and the District of Columbia, the suicide rate was higher for males than for females (23.7 versus 6.1 per 100,000 population). The suicide rate for males was highest for those aged ≥85 years (56.6), whereas for females, the suicide rate was highest for those aged 45–54 years (8.9). In addition, non-Hispanic American Indian or Alaska Native (AI/AN) persons had the highest suicide rates among all racial and ethnic groups (24.3). Among both males and females, the most common method of injury for suicide was a firearm. Among all suicide victims, when circumstances were known (83.5%), suicide was most often preceded by a mental health or substance use–related problem or treatment, suicidal thoughts or plans, a recent or impending crisis, or depressed mood.

The homicide rate was higher for males than for females. Among all homicide victims, the homicide rate was highest among persons aged 20–24 years compared with other age groups. Non-Hispanic Black or African American (Black) males experienced the highest homicide rate of any racial or ethnic group. Among all homicide victims, the most common method of injury was a firearm. When the relationship between a homicide victim and a suspect was known, the suspect was most frequently an acquaintance or friend for male victims and a current or former intimate partner for female victims. Homicide most often was precipitated by an argument or conflict, occurred in conjunction with another crime, or, for female victims, was related to intimate partner violence. Nearly all legal intervention deaths were among males, and the legal intervention death rate was highest among males aged 30–34 years. The legal intervention death rate was highest among AI/AN males, followed by Black males. A firearm was used in most legal intervention deaths. When circumstances were known for legal intervention deaths, the most frequent circumstances reported were the victim used a weapon in the incident and the victim was previously known to authorities.

Other causes of death included unintentional firearm injury deaths and deaths of undetermined intent. Unintentional firearm injury deaths were most frequently experienced by males, non-Hispanic White (White) persons, and persons aged 15–19 years. These deaths most frequently occurred while the shooter was playing with a firearm or were precipitated by a person unintentionally pulling the trigger. The rate of deaths of undetermined intent was highest among males, particularly among AI/AN and Black males, and among adults aged 35–44 years. Poisoning was the most common method in deaths of undetermined intent, and opioids were detected in approximately 70% of decedents tested for those substances.

In Puerto Rico, the homicide rate was 11.5 times higher for males than for females. Firearms were the most common method of injury in homicides (93.6%). When the relationship between the homicide victim and suspect was known, the suspect was most frequently a person known to the victim, but the exact relationship was unclear for male victims and was a current or former intimate partner for female victims. Among male victims, the most common precipitating circumstance was the victim was previously known to authorities (47.1%), whereas among female victims, the most common circumstance was intimate partner violence (29.8%). The suicide rate in Puerto Rico was also higher for males than for females. The most common method for suicide was hanging, strangulation, or suffocation (62.3%). A depressed mood or currently diagnosed mental health problem were frequent circumstances reported for both male and female suicide decedents.

**Interpretation:**

This report provides a detailed summary of data from NVDRS on violent deaths and suicides that occurred in 2022, the first year for which data from all 50 states, the District of Columbia, and Puerto Rico met the NVDRS national data set inclusion criteria. States with large numbers of deaths that meet the NVDRS case definition (California, Florida, and Texas) are moving toward statewide coverage rather than including only a subset of deaths that occurred in their state. The suicide rate was highest among AI/AN and White males, whereas the homicide rate was highest among Black and AI/AN males. Intimate partner violence precipitated a large proportion of homicides among females. Mental health and substance use problems, previous awareness of the victim by authorities, intimate partner problems, interpersonal conflicts, and acute life stressors were primary precipitating circumstances for multiple types of deaths examined. These findings increase the knowledge base about the circumstances associated with these deaths and can assist public health authorities and their partners in developing and informing effective, data-driven approaches to violence prevention.

**Public Health Action:**

The injury-related deaths described in this report are preventable, and data can inform public health action. NVDRS data are used to monitor the occurrence of these fatal injuries and assist public health agencies in developing, implementing, and evaluating programs, policies, and practices to reduce and prevent deaths. States and jurisdictions have used their Violent Death Reporting System data to inform violence prevention efforts and highlight where additional focus is needed. The findings in this report can be used to enhance prevention efforts.

## Introduction

According to National Vital Statistics System (NVSS) mortality data obtained from CDC’s Web-based Injury Statistics Query and Reporting System (WISQARS),* over 81,000 deaths in the United States in 2022 were due to suicide, homicide, legal intervention, or unintentional firearm injuries, or were deaths of undetermined intent that might have been due to violence (*1*). Suicide was the 11th leading cause of death overall in the United States and disproportionately affected non-Hispanic American Indian or Alaska Native (AI/AN) and non-Hispanic White (White) males (*1*). Homicide was the 16th leading cause of death overall in the United States and disproportionately affected young persons and non-Hispanic Black or African American (Black) males (*1*). 

Although NVSS data are a vital resource for surveillance of these deaths, they do not contain detailed information on the circumstances leading up to these deaths, victim-perpetrator relationship, or other characteristics that could be critical for prevention. Public health authorities require accurate, timely, and complete surveillance data to better understand and ultimately prevent the occurrence of these deaths in the United States (*2*–*4*). In 2000, CDC began planning to implement the National Violent Death Reporting System (NVDRS) (*2*) in response to an Institute of Medicine^†^ report noting the need for a national fatal intentional injury surveillance system (*5*). The goals of NVDRS are to

- collect and analyze timely, high-quality data for monitoring the magnitude and characteristics of violent deaths and suicides at national, state, and local levels;
- ensure data are disseminated routinely and expeditiously to public health officials, law enforcement officials, policymakers, and the public;
- ensure data are used to develop, implement, and evaluate programs and strategies that are intended to reduce and prevent violent deaths and suicides at national, state, and local levels; and
- build and strengthen partnerships among organizations and communities at national, state, and local levels to ensure that data are collected and used to reduce and prevent violent deaths and suicides.

NVDRS is a jurisdiction-based active surveillance system that collects data on the characteristics and circumstances associated with violent deaths and suicides among participating states, the District of Columbia, and Puerto Rico (*2*). Deaths collected by NVDRS include suicides, homicides, legal intervention deaths (i.e., deaths caused by law enforcement acting in the line of duty and other persons with legal authority to use deadly force, excluding legal executions), unintentional firearm injury deaths, and deaths of undetermined intent that might have occurred because of violence or suicide.^§^ The term “legal intervention” is a classification incorporated into the *International Classification of Diseases, Tenth Revision,* (ICD-10) (*6*) and does not denote the lawfulness or legality of the circumstances surrounding a death caused by law enforcement.

Before implementation of NVDRS, single data sources (e.g., death certificates) provided only limited information and few circumstances from which to understand patterns of violent deaths and suicides. NVDRS filled this surveillance gap by providing more detailed information. NVDRS is the first system in the United States to 1) provide detailed information on circumstances precipitating these deaths, 2) link multiple source documents (e.g., law enforcement reports and coroner and medical examiner reports) so that each data source can contribute to the study of patterns of these deaths, and 3) link multiple deaths that are related to one another (e.g., multiple homicides, suicide pacts, or homicide followed by suicide of the suspect).

NVDRS data collection began in 2003 with six participating states (Maryland, Massachusetts, New Jersey, Oregon, South Carolina, and Virginia) and has gradually expanded (Figure). Since 2018, CDC has provided NVDRS funding to all 50 states, the District of Columbia, and Puerto Rico. NVDRS data are updated annually and are available to the public through CDC WISQARS - Web-based Injury Statistics Query and Reporting System). Case-level NVDRS data are available to interested researchers who meet eligibility requirements via the NVDRS Restricted Access Database. This report summarizes NVDRS data on violence-related deaths and suicides that occurred in all 50 states, the District of Columbia, and Puerto Rico in 2022. Forty-seven states collected statewide data (Supplementary Box). The three remaining states collected data from a subset of counties in their states (32 California counties, 32 Florida counties, and 13 Texas counties) (Supplementary Box). The NVDRS report for 2021 included data from 48 states, the District of Columbia, and Puerto Rico (*7*); this report includes data from all 50 states for the first time. In addition, whereas previous NVDRS reports presented data on suicides of persons aged ≥10 years, this report includes data on suicides of persons of all ages. The research on suicides of young children is scarce but evolving, and the expansion of the suicide age range addresses the need to further investigate and understand these deaths and how to prevent them (*8*).

**FIGURE:**
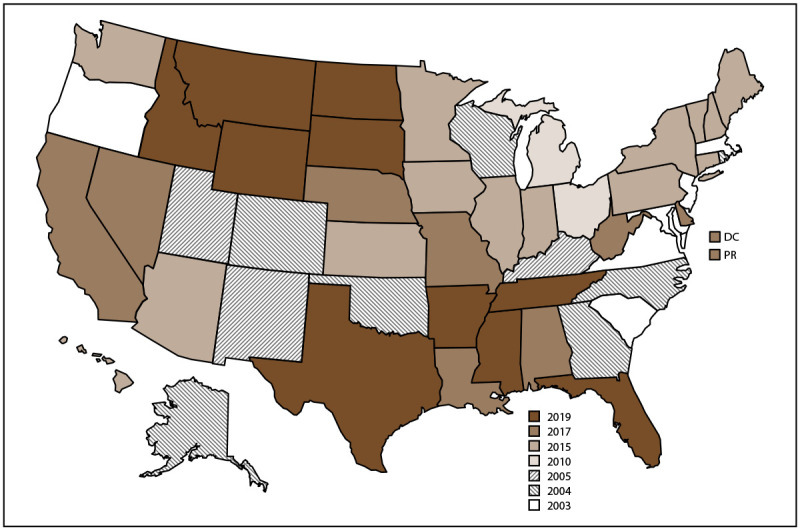
States and jurisdictions participating in the National Violent Death Reporting System, by year of initial data collection* — United States and Puerto Rico, 2003–2022 **Abbreviations:** DC = District of Columbia; NVDRS = National Violent Death Reporting System; PR = Puerto Rico. * Map of United States indicates the year in which the state or jurisdiction began collecting data in NVDRS. Beginning in 2019, all 50 U.S. states, the District of Columbia, and Puerto Rico were participating in the system. California began collecting data for a subset of deaths in 2005 but ended data collection in 2009; however, in 2017, California resumed collecting data for a subset of deaths and expanded coverage in subsequent years. In 2022, California collected data for deaths in 32 counties (Supplementary Box) representing 68% of the state’s population. Michigan collected data for a subset of deaths during 2010–2013 and expanded to collecting statewide data beginning in 2014. In 2015, Illinois, Pennsylvania, and Washington began collecting data on deaths in a subset of counties that represented at least 80% of all deaths in their state or in counties. Washington began collecting statewide data for all deaths beginning in 2018, and Illinois and Pennsylvania began collecting statewide data beginning in 2020. In 2019, Florida and Texas began collecting data for a subset of deaths and expanded coverage in subsequent years. In 2022, Florida collected data for deaths that occurred in 32 counties (Supplementary Box) representing approximately 70% of the state’s population. In 2022, Texas collected data for deaths that occurred in 13 counties (Supplementary Box) representing approximately 63% of the state’s population.

## Methods

NVDRS compiles information from three required data sources: death certificates, coroner or medical examiner records, and law enforcement reports (*2*). Certain participating Violent Death Reporting System (VDRS) programs might also collect information from secondary data sources (e.g., child fatality review team data, Federal Bureau of Investigation Supplementary Homicide Reports, or crime laboratory data). NVDRS combines information for each death and links deaths that are related (e.g., multiple homicides, homicide followed by suicide, or multiple suicides) into a single incident. The ability to analyze linked data can provide a more comprehensive understanding of the type of deaths described in this report. Participating VDRS programs use vital statistics death certificate files or coroner or medical examiner records to identify deaths meeting the NVDRS case definition (see Manner of Death). Each VDRS program reports deaths of residents that occurred within the state, district, or territory (i.e., resident deaths) and those of non-residents who experienced a fatal injury within the state, district, or territory (i.e., occurrent deaths). When a death matching the case definition is identified, NVDRS data abstractors link source documents, link deaths within each incident, code data elements, and write brief narratives of the incident.

In NVDRS, a case is defined as a death resulting from the intentional use of physical force or power, threatened or actual, against oneself, another person, or a group or community (*2*). NVDRS collects information about homicides, suicides, deaths by legal intervention (excluding executions), and deaths of undetermined intent that might have occurred due to violence or suicide. NVDRS also collects information on unintentional firearm injury deaths to provide a more complete picture of firearm-related injury deaths in the United States (see Manner of Death). Cases are included if they are assigned ICD-10 cause of death codes (*6*) aligning with an NVDRS manner of death (Box 1) or a manner of death specified in at least one of the three primary data sources consistent with NVDRS case definitions.

BOX 1 ***International Classification of Diseases, Tenth Revision,* codes used in the National Violent Death Reporting System, 2022**

**Table:** 

**Manner of death**	**Death ≤1 year after injury**	**Death >1 year after injury**	**Death any time after injury**
Intentional self-harm (suicide)	X60–X84	Y87.0	U03 (attributable to terrorism)
Assault (homicide)	X85–X99, Y00–Y09	Y87.1	U01, U02 (attributable to terrorism)
Legal intervention (excluding executions, Y35.5)	Y35.0–Y35.4, Y35.6, Y35.7	Y89.0	Not applicable
Unintentional exposure to inanimate mechanical forces (firearms)	W32–W34	Y86	Not applicable
Event of undetermined intent	Y10–Y34	Y87.2, Y89.9	Not applicable

NVDRS is an incident-based system, and all decedents associated with an incident are grouped in one record. Decisions about whether two or more deaths are related and belong to the same incident are made based on the timing of the injuries rather than on the timing of the deaths. Deaths resulting from injuries that are clearly linked by source documents (e.g., common victims or suspects reported) when the injuries occur within 24 hours of each other are considered part of the same incident. Examples of an incident include 1) a single isolated violent death, 2) two or more related homicides (including legal intervention deaths) in which the fatal injuries were inflicted <24 hours apart, 3) two or more related suicides or deaths of undetermined intent in which the fatal injuries were inflicted <24 hours apart, and 4) a homicide followed by a suicide in which both fatal injuries were inflicted <24 hours apart (*9*).

Information collected from each data source is entered by data abstractors into the NVDRS web-based system (*2*). This system streamlines data abstraction by allowing abstractors to enter data from multiple sources into the same incident record. Internal validation checks, hover-over features that define selected fields, and other quality control measures also are included within the system. Primacy rules and hierarchical algorithms related to the source documents occur at the local VDRS program level. CDC provides access to the web-based system to each VDRS program. VDRS program personnel are provided ongoing training to learn and adhere to CDC guidance regarding the coding of all variables and technical assistance to help increase data quality. Information abstracted into the system is deidentified at the local VDRS program level, and data are transmitted continuously via the web to a CDC-based server. This activity was reviewed by CDC, deemed not research, and was conducted consistent with applicable Federal law and CDC policy.^¶^

### Manner of Death

A manner (i.e., intent) of death for each decedent is assigned by a trained abstractor who integrates information from all source documents. The abstractor-assigned manner of death must be consistent with at least one required data source; typically, all source documents are consistent regarding the manner of death. When a discrepancy exists, the abstractor must assign a manner of death based on a preponderance of evidence in the source documents (*9*); however, such occurrences are rare. For example, if two sources report a death as a suicide and a third reports it as a death of undetermined intent, the death is coded as a suicide.

NVDRS data are categorized into five abstractor-assigned manners of death: 1) suicide, 2) homicide, 3) legal intervention death, 4) unintentional firearm injury death, and 5) death of undetermined intent. The case definitions for each manner of death are described as follows:

- **Suicide.** A suicide is a death resulting from the use of force against oneself when a preponderance of evidence indicates that the use of force was intentional. This category includes the following scenarios: 1) deaths of persons who intended only to injure themselves rather than die by suicide; 2) persons who initially intended to die by suicide and changed their minds but still died as a result of the act; 3) deaths associated with risk-taking behavior without clear intent to inflict a fatal self-injury but associated with high risk for death (e.g., participating in Russian roulette); 4) suicides that occurred while under the influence of substances taken voluntarily; 5) suicides among decedents with mental health problems that affected their thinking, feelings, or mood (e.g., while experiencing an acute episode of a mental health condition, such as schizophrenia or other psychotic conditions, depression, or posttraumatic stress disorder); and 6) suicides involving another person who provided passive (only) assistance to the decedent (e.g., supplying the means or information needed to complete the act). This category does not include deaths caused by chronic or acute substance use without the intent to die, deaths attributed to autoerotic behavior (e.g., self-strangulation during sexual activity), or assisted suicides (legal or nonlegal). Corresponding ICD-10 codes included in NVDRS are X60–X84, Y87.0, and U03 (Box 1).
- **Homicide.** A homicide is a death resulting from the use of physical force or power, threatened or actual, against another person, group, or community when a preponderance of evidence indicates that the use of force was intentional. Two special scenarios that CDC’s National Center for Health Statistics regards as homicides are included in the NVDRS case definition: 1) arson with no specified intent to injure someone and 2) a stabbing with intent unspecified. This category also includes the following scenarios: 1) deaths when the suspect intended to only injure rather than kill the victim, 2) deaths resulting from a heart attack induced when the suspect used force or power against the victim, 3) deaths that occurred when a person killed an attacker in self-defense, 4) deaths resulting from a weapon that discharged unintentionally while being used to control or frighten the victim, 5) deaths attributed to child abuse regardless of intent, 6) deaths attributed to an intentional act of neglect by one person against another, 7) deaths of liveborn infants that resulted from a direct injury because of violence sustained before birth, and 8) deaths identified as a justifiable homicide when the person committing homicide was not a law enforcement officer. This category excludes vehicular homicide without intent to injure, unintentional poisoning deaths involving illegal or prescription drugs even when the person who provided drugs was charged with homicide, unintentional firearm injury deaths (a separate category in NVDRS), combat deaths or acts of war, deaths of unborn fetuses, and deaths of infants that resulted indirectly from violence sustained by the mother before birth (e.g., death from prematurity after premature labor brought on by violence). Corresponding ICD-10 codes included in NVDRS are X85–X99, Y00–Y09, Y87.1, and U01–U02 (Box 1).
- **Legal intervention.** A death from legal intervention is a death in which a person is killed or died as a result of injuries inflicted by a law enforcement officer or another peace officer (i.e., a person with specified legal authority to use deadly force), including military police, while acting in the line of duty. The term “legal intervention” is a classification from ICD-10 (Y35.0) and does not denote the lawfulness or legality of the circumstances surrounding a death caused by law enforcement. Legal intervention deaths also include a small subset of cases in which force was applied without clear lethal intent (e.g., during restraint or when applying force with a typically non-deadly weapon, such as a Taser) or in which the death occurred while the person was fleeing capture. This category excludes legal executions. Corresponding ICD-10 codes included in NVDRS are Y35.0–Y35.4, Y35.6, Y35.7, and Y89.0 (Box 1).
- **Unintentional firearm injury.** An unintentional firearm injury death is a death resulting from a penetrating injury or gunshot wound from a weapon that uses a powder charge to fire a projectile and for which a preponderance of evidence indicates that the shooting was not directed intentionally at the decedent with an intent to injure. Examples include the following: 1) a person who received a self-inflicted wound while playing with a firearm; 2) a person who mistakenly believed a firearm was unloaded and shot another person; 3) a child aged <6 years who shot himself or herself (and was not determined to be a suicide) or another person; 4) a person who died as a result of a celebratory firing that was not intended to frighten, control, or harm anyone; 5) a person who unintentionally shot himself or herself when using a firearm to frighten, control, or harm another person; 6) a soldier who was shot during a field exercise but not in a combat situation; and 7) an infant who died after birth from an unintentional firearm injury that was sustained in utero. This category excludes injuries caused by unintentionally striking a person with the firearm (e.g., hitting a person on the head with the firearm rather than firing a projectile) and unintentional injuries from non-powder guns (e.g., BB, pellet, or other compressed air-powered or compressed gas-powered guns). Corresponding ICD-10 codes included in NVDRS are W32–W34 and Y86 (Box 1).
- **Undetermined intent.** A death of undetermined intent is a death resulting from the use of force or power against oneself or another person for which the evidence indicating one manner of death is no more compelling than evidence indicating another. This category includes coroner or medical examiner rulings in which records from data providers indicate that investigators did not find enough evidence to determine whether the injury was intentional (e.g., unclear whether a drug overdose was unintentional or a suicide). Corresponding ICD-10 codes included in NVDRS are Y10–Y34, Y87.2, and Y89.9 (Box 1).

### Variables Analyzed

NVDRS collects hundreds of unique variables for each death (Boxes 2 and 3). The number of variables recorded for each incident depends on the content and completeness of the source documents. Variables in NVDRS include

BOX 2 **Methods used to inflict injury — National Violent Death Reporting System, 2022**

- Firearm: method that uses a powder charge to fire a projectile from the weapon (excludes BB gun, pellet gun, or compressed air- or gas-powered gun)
- Hanging, strangulation, or suffocation (e.g., hanging by the neck, manual strangulation, or plastic bag over the head)
- Poisoning (e.g., fatal ingestion or injection of an illegal drug, alcohol, pharmaceutical, carbon monoxide, gas, rat poison, or insecticide)
- Sharp instrument (e.g., knife, razor, machete, or pointed instrument)
- Blunt instrument (e.g., club, bat, rock, or brick)
- Fall: being pushed or jumping
- Motor vehicle (e.g., car, bus, motorcycle, or other transport vehicle)
- Other transport vehicle (e.g., train, plane, or boat)
- Personal weapons (e.g., hands, fists, or feet)
- Drowning: inhalation of liquid (e.g., in bathtub, lake, or other source of water or liquid)
- Fire or burns: inhalation of smoke or the direct effects of fire or chemical burns
- Shaking (e.g., shaken baby syndrome)
- Intentional neglect: starvation, lack of adequate supervision, or withholding of health care
- Explosive (e.g., bomb, rocket, or grenade)
- Non-powder gun (e.g., BB, pellet, or compressed air- or gas-powered gun)
- Biologic weapons (e.g., anthrax, plague, or botulism)
- Other (single method): any method other than those already listed (e.g., electrocution or exposure to environment or weather)
- Unknown: method not reported or not known

BOX 3 Circumstances preceding* fatal injury, by manner of death — National Violent Death Reporting System, 2022

**All Manners of Death**

*Mental Health and Substance Us*e

- Alcohol problem: decedent was perceived by self or others to have a problem with, or to be addicted to or dependent on, alcohol.
- Current depressed mood: decedent was perceived by self or others to be feeling depressed at the time of incident.
- Current diagnosed mental health problem: decedent was identified as having a mental health disorder or syndrome listed in the *Diagnostic and Statistical Manual, Version 5* (DSM-5), with the exception of alcohol and other substance dependence (these are captured in separate variables).
- Current mental health or substance use treatment: decedent was receiving mental health or substance use treatment as evidenced by a current prescription for a psychotropic medication, visit or visits to a mental health professional, or participation in a therapy group or outpatient program within the previous 2 months.
- History of ever being treated for mental health or substance use problem: decedent was identified as having ever received mental health or substance use treatment.
- Non-adherence to treatment for a mental health or substance use problem: decedent did not actively participate in a prescribed regimen for their mental health or substance use treatment or did not follow a set treatment plan as recommended by a mental health or medical professional.
- Other substance use problem (excludes alcohol): decedent was perceived by self or others to have a problem with, or be addicted to or dependent on, a substance other than alcohol.
- Other addiction: decedent was perceived by self or others to have an addiction to or dependency on something other than alcohol or other substance (e.g., gambling or sex).
- Type of mental health diagnosis: type of DSM-5 diagnosis reported for the decedent.

*Crime and Criminal Activity*

- Precipitated by another crime: incident occurred as the result of another serious crime.
- Crime in progress: another serious or felony-related crime was in progress at the time of the incident.
- Nature of crime: the specific type of other crime that occurred precipitated the incident (e.g., sexual assault, gambling, robbery, or drug trafficking).

*Relationship and Life Stressors*

- Argument or conflict: a specific argument or disagreement led to the victim’s death.
- Caretaker abuse or neglect led to death: decedent was experiencing physical, sexual, or psychological abuse; physical (including medical or dental), emotional, or educational neglect; exposure to a violent environment; or inadequate supervision by a caretaker that led to death.
- Exposure to disaster: decedent was exposed to a disaster (e.g., earthquake, bombing, or COVID-19 pandemic) that was perceived as contributing to the incident.
- Family relationship problem: decedent was experiencing a problem with a family member other than an intimate partner.
- Family stressor: decedent was experiencing a problem related to the family home environment that was not related to relationship problems and involved family members other than intimate partners.
- History of child abuse or neglect: as a child, decedent had history of physical, sexual, or psychological abuse; physical (including medical or dental), emotional, or educational neglect; exposure to a violent environment; or inadequate supervision by a caretaker.
- Household known to local authorities: someone in the household, other than the decedent, had previous contact with local authorities.
- Living transition or loss of independent living: decedent recently transitioned from an independent or family living situation (e.g., family home or living on one’s own) to an assisted one, or such a transition was imminent.
- Other relationship problem (non-intimate): decedent was experiencing a problem with a friend or associate (other than an intimate partner or family member).
- Perpetrator of interpersonal violence during previous month: decedent perpetrated interpersonal violence during the past month.
- Physical fight (two persons, not a brawl): a physical fight between two persons that resulted in the death of the decedent, who was either involved in the fight, a bystander, or trying to stop the fight.
- Victim known to authorities: decedent had a history of contact with or was otherwise known to local, state, Federal, or international authorities. “Authorities” encompasses anyone who is in a position of authority (e.g., law enforcement, emergency medical services, child protective services, public safety officer, or judge) who has the power or right to give orders, make decisions, and/or enforce obedience. Being “known” to authorities might or might not involve direct contact on the part of the victim.
- Victim of interpersonal violence during previous month: decedent was the target of interpersonal violence during the past month.

*Child Victim Incident*

- Previous Child Protective Services report on the child victim’s household: a Child Protective Services report was filed on the child decedent’s household before the fatal incident.
- Substance use problems in child victim’s household: evidence of substance use or misuse in child decedent’s household.

*Crisis Circumstances*

- Crisis during previous or upcoming 2 weeks: current crisis or acute precipitating event or events that either occurred during the previous 2 weeks or was impending in the following 2 weeks (e.g., a trial for a criminal offense begins the following week and appeared to have contributed to the death). Crises are associated with specific circumstance variables (e.g., alcohol problem was a crisis, or a family relationship problem was a crisis).
- Other crisis: a crisis related to a death but not captured by any of the standard circumstances.

**Suicide or Death of Undetermined Intent**

*Interpersonal Factors*

- Intimate partner problem: decedent was experiencing a problem with a current or former intimate partner.
- Suicide of family member or friend: decedent was distraught over, or reacting to, the suicide of a family member or friend.
- Other death of family member or friend: decedent was distraught over, or reacting to, the non-suicide death of a family member or friend.

*Life Stressors*

- Caregiver burden: stress or burden perceived by the decedent as a caregiver of a chronically ill, disabled, or elderly person appears to have contributed to the death.
- Eviction or loss of housing: decedent was experiencing a recent or impending eviction or other loss of housing, or the threat of eviction or loss of housing.
- Financial problem: decedent was experiencing a financial problem (e.g., bankruptcy, overwhelming debt, or foreclosure of a home or business).
- History of traumatic brain injury: decedent had history of traumatic brain injury.
- Job problem: decedent was either experiencing a problem at work or was having a problem with joblessness.
- Non-criminal legal problem: decedent was facing a civil legal problem (e.g., a child custody or civil lawsuit).
- Physical health problem: decedent was experiencing a physical health problem (e.g., a recent cancer diagnosis or chronic pain).
- Recent criminal legal problem: decedent was facing a criminal legal problem (e.g., recent or impending arrest or upcoming criminal court date).
- School problem: decedent was experiencing a problem related to school (e.g., poor grades, bullying, social exclusion at school, or performance pressures).
- Traumatic anniversary: the incident occurred on or near the anniversary of a traumatic event in the decedent’s life.

*Suicide and Self-Harm Event*

- History of attempting suicide: decedent had previously attempted suicide before the fatal incident.
- History of non-suicidal self-injury or self-harm: decedent had a history of intentionally inflicting pain or injuring one’s own body without the conscious intent of dying by suicide.
- History of suicidal thoughts or plans: decedent had previously expressed suicidal thoughts or plans.
- Left a suicide note: decedent left a note, email message, video, or other communication indicating intent to die by suicide.

*Suicide Disclosure*

- Disclosed suicidal intent: decedent had recently expressed suicidal feelings to another person with time for that person to intervene.
- Disclosed intent to whom: type of person (e.g., family member or current or former intimate partner) to whom the decedent recently disclosed suicidal thoughts or plans.

**Homicide or Legal Intervention Death**

*Interpersonal Factors*

- Intimate partner violence–related: incident is related to conflict between current or former intimate partners; includes all deaths of an intimate partner as well as others (e.g., child, parent, friend, or law enforcement officer) killed in an incident that originated in a conflict between intimate partners.
- Jealousy (lovers’ triangle): jealousy or distress over an intimate partner’s relationship or suspected relationship with another person.

*Crime and Criminal Activity*

- Drug involvement: drug dealing, drug trade, or illegal drug use suspected to have played a role in precipitating the incident.
- Gang-related: motive of the incident was gang-related, or a gang member was a suspect or decedent in the incident.

*Incident Events*

- Brawl: mutual physical fight involving three or more persons.
- Drive-by shooting: suspect drove near the decedent and fired a weapon while driving or stepped out of the car just long enough to use a weapon.
- Hate crime: decedent was selected intentionally because of his or her actual or perceived sex, religion, sexual orientation, race, ethnicity, disability, immigrant status, or national origin.
- Justifiable self-defense: decedent was killed by a law enforcement officer in the line of duty or by a civilian in legitimate self-defense or in defense of others.
- Mentally ill suspect: suspect’s attack on decedent was believed to be the direct result of a mental health problem (e.g., schizophrenia or other psychotic condition, depression, or posttraumatic stress disorder).
- Mercy killing: decedent wished to die because of a terminal or hopeless disease or condition, and documentation indicates that the decedent wanted to be killed.
- Prostitution: prostitution or related activity that includes prostitutes, pimps, clients, or others involved in such activity.
- Random violence: decedent was killed in a random act of violence (i.e., an act in which the suspect is not concerned with who is being harmed, just that someone is being harmed).
- Stalking: pattern of unwanted harassing or threatening tactics by either the decedent or suspect.
- Victim used a weapon: decedent used a weapon to attack or defend during the course of the incident.
- Victim was a bystander: decedent was not the intended target in the incident (e.g., pedestrian walking past a gang fight).
- Victim was an intervener assisting a crime victim: decedent was attempting to assist a crime victim at the time of the incident (e.g., a child attempts to intervene and is killed while trying to assist a parent who is being assaulted).
- Victim was a police officer on duty: decedent was a law enforcement officer killed in the line of duty.
- Walk-by assault: decedent was killed by a targeted attack (e.g., ambush) where the suspect fled on foot.

*Child Victim Homicide Incident*

- Caregiver use of corporal punishment: corporal punishment (i.e., physical punishment with or without an implement that is intended to punish or discipline a child) by the child’s caregiver contributed to the death of the child decedent.

**Unintentional Firearm Injury Death**

*Context of Injury*

- Celebratory firing: shooter fired firearm in celebratory manner (e.g., firing into the air at midnight on New Year’s Eve).
- Cleaning firearm: shooter pulled trigger or firearm discharged while cleaning, repairing, assembling, or disassembling firearm.
- Hunting: death occurred any time after leaving home for a hunting trip and before returning home from a hunting trip.
- Loading or unloading firearm: firearm discharged when the shooter was loading or unloading ammunition.
- Playing with firearm: shooter was playing with a firearm when it discharged.
- Showing firearm to others: firearm was being shown to another person when it discharged, or the trigger was pulled.
- Target shooting: shooter was aiming for a target and unintentionally hit the decedent; can be at a shooting range or an informal backyard setting (e.g., teenagers shooting at signposts on a fence).
- Other context of injury: shooting occurred during some context other than those already described.

*Mechanism of Injury*

- Bullet ricocheted: bullet ricocheted from its intended target and struck the decedent.
- Firearm fired due to defect or malfunction: firearm had a defect or malfunctioned as determined by a trained firearm examiner.
- Firearm fired while holstering: firearm was being replaced or removed from holster or clothing.
- Firearm fired while operating safety or lock: shooter unintentionally fired the firearm while operating the safety or lock.
- Firearm was dropped: firearm discharged when it was dropped.
- Firearm was mistaken for toy: firearm was mistaken for a toy and was fired without the user understanding the danger.
- Thought firearm safety was engaged: shooter thought the safety was on and firearm would not discharge.
- Thought firearm was unloaded, magazine disengaged: shooter thought the firearm was unloaded because the magazine was disengaged.
- Thought firearm was unloaded, other reason: shooter thought the firearm was unloaded for reason other than magazine disengaged or for an unspecified reason.
- Unintentionally pulled trigger: shooter unintentionally pulled the trigger (e.g., while grabbing the firearm or holding it too tightly).
- Other mechanism of injury: shooting occurred as the result of a mechanism not already described.

* Circumstances preceding death are defined as the events that precipitated, occurred during, or otherwise contributed to the infliction of a fatal injury as identified by investigators.

- manner of death (i.e., the intent to cause death [suicide, homicide, legal intervention, unintentional firearm, and undetermined] of the person on whom a fatal injury was inflicted);
- demographic information (e.g., age, sex, and race and ethnicity) of victims and suspects (if applicable);
- method of injury (i.e., the mechanism used to inflict a fatal injury) (Box 2);
- location, date, and time of injury and death;
- toxicology findings for decedents who were tested (findings were considered positive if the substance’s toxicology result was labeled as “positive,” “present,” or “presumptive presence”; had a numeric level greater than zero; or had any similar indication in the source documents that any detectable level of the substance was found);
- circumstances (i.e., the events that preceded, precipitated, or occurred during or otherwise contributed to the fatal incident as identified by investigators as relevant and therefore might have contributed to the infliction of a fatal injury) (Box 3);
- whether the decedent was a victim (i.e., a person who died as a result of a suicide or violence-related injury) or both a suspect and a victim (i.e., a person believed to have inflicted a fatal injury on a victim who then was fatally injured, such as the perpetrator of a homicide followed by suicide);
- information about any known suspects (i.e., a person or persons believed to have inflicted a fatal injury on a victim);
- incident (i.e., an occurrence in which one or more persons sustained a fatal injury that was linked to a common event or perpetrated by the same suspect or suspects during a 24-hour period); and
- type of incident (i.e., a combination of the manner of death and whether single or multiple victims were involved in an incident).

### Circumstances Preceding Death

Circumstances preceding death are defined as the events that precipitated, occurred during, or otherwise contributed to the infliction of a fatal injury as identified by investigators (Box 3). Circumstances are reported based on the content of coroner or medical examiner and law enforcement investigative reports. Certain circumstances are coded to a specific manner of death (e.g., “history of suicidal thoughts or plans” is collected for suicides and deaths of undetermined intent); other circumstances are coded across all manners of death (e.g., “ever treated for mental health or substance use problem”). The data abstractor reviews a list of potential circumstances and is required to code all circumstances that are known to relate to each incident. If circumstances are unknown (e.g., a body found in the woods with no other details reported), the data abstractor does not endorse circumstances; these deaths are then excluded from the denominator for circumstance values. If either the coroner or medical examiner report or law enforcement report indicates the presence of a circumstance, then the abstractor endorses the circumstance. For example, if a law enforcement report indicates that a decedent had disclosed thoughts of suicide or an intent to die by suicide, then the circumstance variable “recent disclosure of suicidal thoughts or intent” is endorsed.

Data abstractors draft two incident narratives: one that summarizes the sequence of events of the incident from the perspective of the coroner or medical examiner record and one that summarizes the sequence of events of the incident from the perspective of the law enforcement report. In addition to briefly summarizing the incident (i.e., including the who, what, when, where, and why of the incident), the narratives provide supporting information, context, and details on circumstances indicated by the data abstractor for understanding the incident; record information and additional detail that cannot be captured elsewhere; and facilitate data quality control checks on the coding of key variables.

### Coding Training and Quality Control

Ongoing coding support for data abstractors is provided by CDC through an electronic help desk, monthly conference calls, annual in-person or virtual meetings that include coding training for data abstractors, and regular technical assistance conference calls with individual VDRS programs. In addition, all data abstractors are invited to participate in monthly coding work group calls. VDRS programs can conduct additional abstractor training workshops and activities at their own discretion, including through the use of NVDRS Data Abstractor eLearn Training Modules. An NVDRS coding manual (*9*) with CDC-issued standard guidance on coding criteria and examples for each data element is provided to each VDRS program and is publicly available (NVDRS Web Coding Manual Version 6.1). Software features that enhance coding reliability include automated validation rules and a hover-over feature containing variable-specific information.

Each year, VDRS programs are required to reabstract at least 5% of cases using multiple abstractors to identify inconsistencies. In addition, each VDRS program’s data quality plan is evaluated by CDC. Before the data are released each year, CDC conducts a quality control analysis that involves the review of multiple variables for data inconsistencies, with special focus on abstractor-assigned variables (e.g., method of injury and manner of death). If CDC finds inconsistencies, the VDRS program is notified and asked for a response or correction. VDRS programs must meet CDC standards for completeness of circumstance data to be included in the national data set. VDRS programs must have circumstance information abstracted from either the coroner or medical examiner record or the law enforcement report for at least 50% of deaths. However, VDRS programs often exceed this requirement. In addition, core variables that represent demographic characteristics (e.g., age, sex, and race and ethnicity) and manners of death were missing or unknown for <0.3% of cases in 2022. To ensure the final data set has no duplicate records, during the data closeout process, NVDRS identifies any records within VDRS programs that match on a subset of 14 key variables and asks VDRS programs to review these records to determine whether they are true duplicates. One record in any set of two or more records that are true duplicates is retained, and the others are deleted by the VDRS program. Next, NVDRS uses SAS software (version 9.4; SAS Institute) to search for any instances of duplicates of a unique identification variable associated with each decedent record. As a third and final check for duplicates, the SAS data set is created with an index that only executes successfully if no duplicates of this identification variable are found.

### Time Frame

VDRS programs are required to begin entering each death into the web-based system within 4 months from the date the death occurred. This report focuses on deaths occurring in 2022. Deaths of multiple victim incidents are included if the first death of the incident occurred in 2022. VDRS programs then have 16 months from the end of the calendar year in which the death occurred to complete each incident record. Although VDRS programs typically meet timeliness requirements, additional details about an incident occasionally arrive after a deadline has passed. New incidents might also be identified after the deadline (e.g., when a death certificate is revised, new evidence is obtained that changes a manner of death, or an ICD-10 misclassification is corrected to meet the NVDRS case definition). These additional data are incorporated on an ongoing basis into NVDRS when analysis files are updated in real time in the web-based system; historically, during the 6 months after the data collection period for a data year, the case counts have increased by <0.1%.

### Inclusion Criteria

The inclusion criteria for deaths in this report are as follows: 1) cases met the NVDRS case definition; 2) cases occurred in states and jurisdictions participating in NVDRS in 2022; and 3) at least 50% of cases for each included state, district, territory, or subset of counties had circumstance information collected from the coroner or medical examiner record or law enforcement report.

Of the participating VDRS programs, 47 states (Supplementary Box) collected information on all deaths that met the case definition and occurred in their state in 2022. In addition, data were collected on all deaths that met the case definition that occurred in the District of Columbia and Puerto Rico in 2022. 

Three states (California, Florida, and Texas) joined NVDRS with plans to collect data on deaths in a subset of counties. California collected data from death certificates on all deaths that met the case definition in the state in 2022 (n = 7,002); data for deaths that occurred in 32 counties (Supplementary Box) also included information from coroner or medical examiner records and law enforcement reports and are included throughout the rest of the report (n = 4,519 [64.5%]). These 32 counties represented 67.7% of California’s 2022 population (*10*). 

Florida collected data from death certificates on all deaths that met the case definition in the state in 2022 (n = 5,162); data for deaths that occurred in 32 counties (Supplementary Box) also included information from coroner or medical examiner records and law enforcement reports and are included throughout the rest of the report (n = 3,434 [66.5%]). These 32 counties represented 70.4% of Florida’s 2022 population (*10*). 

Texas also collected data from death certificates on all deaths that met the case definition in the state in 2022 (n = 7,054); data for deaths that occurred in 13 counties (Supplementary Box) also included information from coroner or medical examiner records and law enforcement reports and are included throughout the rest of the report (n = 4,396 [62.3%]). These 13 counties represented 63.0% of the state’s 2022 population (*10*). Deaths from California, Florida, and Texas that only had data from death certificates are available (Supplementary Table 2). For all other analyses involving California, Florida, and Texas, only deaths from the counties listed were included. Because <100% of deaths were abstracted, data from California, Florida, and Texas do not represent all cases occurring in these states.

### Analyses

This report includes data for deaths that occurred in 50 states (47 states collecting statewide data, 32 California counties, 32 Florida counties, and 13 Texas counties), the District of Columbia, and Puerto Rico in 2022. VDRS program-level data received by CDC as of June 28, 2024, were consolidated and analyzed. The numbers, percentages, and crude rates are presented in aggregate for all deaths by the abstractor-assigned manner of death. The rates could not be calculated for certain variables (e.g., circumstances) because denominators were unknown. The rates for cells with frequency <20 are not reported because of the instability of those rates. Denominators for the rates for the three states that did not collect statewide data (California, Florida, and Texas) correspond to the populations of the counties from which data were collected. 

The U.S. Census Bureau’s county-level population estimates for 2022 were used as denominators in the crude rate calculations for the 50 states (47 states collecting statewide data, 32 California counties, 32 Florida counties, and 13 Texas counties) and the District of Columbia (*11*). Data for Puerto Rico were analyzed separately because the rates specific to race and ethnicity are not available for Puerto Rico; the U.S. Census Bureau estimates for Puerto Rico do not include race or Hispanic or Latino (Hispanic) origin (*12*). Population estimates by sex and age were used as denominators in the crude rate calculations for Puerto Rico (*13*).

## Results

### Deaths in 50 States and the District of Columbia

For 2022, a total of 50 states (47 states collecting statewide data, 32 California counties, 32 Florida counties, and 13 Texas counties) and the District of Columbia collected NVDRS data on 72,127 incidents involving 74,148 deaths (Supplementary Table 1). Suicides (n = 44,917 [60.6%]) accounted for the highest rate of deaths captured by NVDRS (14.8 per 100,000 population). The homicide rate was 7.4 per 100,000 population (n = 22,395 [30.2%]). Deaths of undetermined intent (n = 5,292 [7.1%]), legal intervention deaths (n = 1,014 [1.4%]), and unintentional firearm injury deaths (n = 530 [<1.0%]) occurred at lower rates (1.7, 0.3, and 0.2, respectively). Data for deaths by manner that include statewide counts and the rates for California, Florida, and Texas are available (Supplementary Table 2). More than half of NVDRS deaths involved firearms as the method of injury (57.1%), and the majority of victims (61.3%) were injured in a house or apartment (Supplementary Table 3). A total of 79.4% of suicides, homicides, and legal intervention deaths had circumstance data from either the coroner or medical examiner record or the law enforcement report (data not shown).

### Suicides

#### Sex, Age Group, and Race and Ethnicity

For 2022, a total of 50 states (47 states collecting statewide data, 32 California counties, 32 Florida counties, and 13 Texas counties) and the District of Columbia collected NVDRS data on 44,880 incidents involving 44,917 suicides (Supplementary Table 1). The overall suicide rate was 14.8 per 100,000 population (Table 1).

**TABLE 1:** Number, percentage,* and rate^†^ of suicides, by decedent’s selected demographics,^§^ method of injury, location of injury, and incident characteristics — National Violent Death Reporting System, 50 states^¶^ and District of Columbia, 2022

Characteristic	Male		Female		Total	
	No. (%)	Rate	No. (%)	Rate	No. (%)	Rate
**Age group, yrs**						
<10	8 (<1.0)	—**	1 (<1.0)	—	9 (<1.0)	—
10–14	286 (<1.0)	3.0	165 (1.8)	1.8	452 (1.0)	2.4
15–19	1,455 (4.1)	14.5	473 (5.1)	5.0	1,929 (4.3)	9.9
20–24	2,825 (7.9)	26.9	686 (7.3)	6.8	3,512 (7.8)	17.1
25–29	2,982 (8.4)	28.9	744 (8.0)	7.5	3,728 (8.3)	18.4
30–34	3,326 (9.4)	30.9	862 (9.2)	8.2	4,188 (9.3)	19.7
35–44	5,898 (16.6)	29.3	1,584 (17.0)	8.1	7,482 (16.7)	18.8
45–54	5,440 (15.3)	29.4	1,641 (17.6)	8.9	7,081 (15.8)	19.2
55–64	5,533 (15.6)	29.3	1,604 (17.2)	8.2	7,137 (15.9)	18.6
65–74	3,916 (11.0)	27.0	996 (10.7)	6.1	4,912 (10.9)	15.9
75–84	2,726 (7.7)	38.9	430 (4.6)	4.8	3,156 (7.0)	19.9
≥85	1,169 (3.3)	56.6	156 (1.7)	4.1	1,325 (2.9)	22.4
Unknown	6 (<1.0)	—	0 (—)	—	6 (<1.0)	—
**Race and ethnicity^††^**						
American Indian or Alaska Native	410 (1.2)	36.2	148 (1.6)	12.8	558 (1.2)	24.3
Asian	894 (2.5)	10.1	397 (4.2)	4.2	1,291 (2.9)	7.0
Black or African American	2,859 (8.0)	15.1	710 (7.6)	3.5	3,569 (7.9)	9.1
Native Hawaiian or other Pacific Islander	71 (<1.0)	24.5	13 (<1.0)	—	84 (<1.0)	14.7
White	27,258 (76.6)	30.1	7,018 (75.1)	7.6	34,280 (76.3)	18.8
More than one race	467 (1.3)	12.8	151 (1.6)	4.1	619 (1.4)	8.5
Hispanic or Latino	3,421 (9.6)	12.9	861 (9.2)	3.3	4,282 (9.5)	8.2
Unspecified or unknown	190 (<1.0)	—	44 (<1.0)	—	234 (<1.0)	—
**Method of injury**						
Firearm	20,544 (57.8)	13.7	3,057 (32.7)	2.0	23,601 (52.5)	7.8
Hanging, strangulation, or suffocation	8,624 (24.2)	5.7	2,511 (26.9)	1.6	11,137 (24.8)	3.7
Poisoning	2,474 (7.0)	1.6	2,598 (27.8)	1.7	5,074 (11.3)	1.7
Fall	889 (2.5)	0.6	305 (3.3)	0.2	1,194 (2.7)	0.4
Sharp instrument	769 (2.2)	0.5	160 (1.7)	0.1	929 (2.1)	0.3
Motor vehicle (e.g., bus, motorcycle, or other transport vehicle)	515 (1.4)	0.3	173 (1.9)	0.1	688 (1.5)	0.2
Drowning	263 (<1.0)	0.2	133 (1.4)	<0.1	396 (<1.0)	0.1
Fire or burns	123 (<1.0)	<0.1	38 (<1.0)	<0.1	161 (<1.0)	<0.1
Blunt instrument	78 (<1.0)	<0.1	24 (<1.0)	<0.1	102 (<1.0)	<0.1
Other (e.g., Taser, electrocution, nail gun, intentional neglect, or personal weapons)	60 (<1.0)	—	16 (<1.0)	—	76 (<1.0)	—
Unknown	1,231 (3.5)	—	327 (3.5)	—	1,559 (3.5)	—
**Location of injury**						
House or apartment	24,907 (70.0)	16.6	7,202 (77.1)	4.7	32,111 (71.5)	10.6
Motor vehicle	2,005 (5.6)	1.3	356 (3.8)	0.2	2,363 (5.3)	0.8
Natural area	1,594 (4.5)	1.1	301 (3.2)	0.2	1,895 (4.2)	0.6
Street or highway	990 (2.8)	0.7	219 (2.3)	0.1	1,209 (2.7)	0.4
Hotel or motel	755 (2.1)	0.5	277 (3.0)	0.2	1,032 (2.3)	0.3
Park, playground, or sports or athletic area	637 (1.8)	0.4	82 (<1.0)	<0.1	719 (1.6)	0.2
Parking lot, public garage, or public transport	594 (1.7)	0.4	106 (1.1)	<0.1	700 (1.6)	0.2
Jail or prison	599 (1.7)	0.4	55 (<1.0)	<0.1	654 (1.5)	0.2
Commercial or retail area	416 (1.2)	0.3	59 (<1.0)	<0.1	475 (1.1)	0.2
Supervised residential facility	249 (<1.0)	0.2	86 (<1.0)	<0.1	335 (<1.0)	0.1
Bridge	255 (<1.0)	0.2	68 (<1.0)	<0.1	323 (<1.0)	0.1
Railroad tracks	213 (<1.0)	0.1	72 (<1.0)	<0.1	285 (<1.0)	<0.1
Hospital or medical facility	141 (<1.0)	<0.1	37 (<1.0)	<0.1	178 (<1.0)	<0.1
Other location^§§^	1,304 (3.7)	—	199 (2.1)	—	1,503 (3.3)	—
Unknown	911 (2.6)	—	223 (2.4)	—	1,135 (2.5)	—
**Incident characteristic**						
Emergency medical services present	22,761 (64.0)	15.2	6,347 (67.9)	4.2	29,112 (64.8)	9.6
Injured at victim’s home	22,073 (62.1)	14.7	6,430 (68.8)	4.2	28,505 (63.5)	9.4
Victim was suspected of alcohol use preceding the incident	5,386 (15.1)	3.6	1,347 (14.4)	0.9	6,733 (15.0)	2.2
Victim was recently released from an institutional setting	2,031 (5.7)	1.4	577 (6.2)	0.4	2,609 (5.8)	0.9
Child present or witnessed incident	1,538 (4.3)	1.0	484 (5.2)	0.3	2,023 (4.5)	0.7
Victim was in public custody when injury occurred	1,098 (3.1)	0.7	107 (1.1)	<0.1	1,205 (2.7)	0.4
Victim was experiencing housing instability	915 (2.6)	0.6	237 (2.5)	0.2	1,152 (2.6)	0.4
Victim was experiencing homelessness	572 (1.6)	0.4	102 (1.1)	<0.1	674 (1.5)	0.2
Victim was injured at work or while working	364 (1.0)	0.2	28 (<1.0)	<0.1	392 (<1.0)	0.1
**Total**	**35,570 (100)**	**23.7**	**9,342 (100)**	**6.1**	**44,917 (100)**	**14.8**

* Percentages might not total 100% due to rounding.

^†^ Per 100,000 population.

^§^ Sex was unknown for five decedents.

^¶^ Data for California are for deaths that occurred in 32 counties, data for Florida are for deaths that occurred in 32 counties, and data for Texas are for deaths that occurred in 13 counties (Supplementary Box). Denominators for the rates for California, Florida, and Texas represent only the populations of the countries from which the data were collected.

** Dashes indicate cell data are suppressed because number of decedents is <20 or characteristic response is “Other” or “Unknown.”

^††^ Persons of Hispanic or Latino (Hispanic) origin might be of any race but are categorized as Hispanic; all racial groups are non-Hispanic.

^§§^ Other location includes (in descending order): industrial or construction area; preschool, school, college, or school bus; farm; cemetery, graveyard, or other burial ground; office building; synagogue, church, or temple; abandoned house, building, or warehouse; bar or nightclub; and other unspecified location.

The overall suicide rate for males (23.7 per 100,000 population) was 3.9 times the rate for females (6.1) (Table 1). The suicide rate for males ranged from 1.7 to 13.8 times the rate for females across age groups and 2.4 to 4.3 times the rate for females across racial and ethnic groups. Adults aged ≥85 years (22.4), 75–84 years (19.9), and 30–34 years (19.7) had the highest rates of suicide across age groups. White persons accounted for a majority (76.3%) of suicides; however, AI/AN persons had the highest rate of suicide (24.3) among all racial and ethnic groups.

By age group, males aged ≥85 years had the highest rate of suicide (56.6 per 100,000 population), followed by males aged 75–84 years (38.9) and 30–34 years (30.9) (Table 1). Across racial and ethnic groups, AI/AN males had the highest rate of suicide (36.2), followed by White males (30.1) and non-Hispanic Native Hawaiian or other Pacific Islander (NH/PI) males (24.5). The rate of suicide for AI/AN males was 3.6 times the rate for males with the lowest rate (i.e., non-Hispanic Asian [Asian]) (10.1). The suicide rate was 15.1 for Black males, 12.8 for males of more than one race, and 12.9 for Hispanic males.

Females aged 45–54 years had the highest rate of suicide (8.9 per 100,000 population), followed by those aged 30–34 years and 55–64 years (both 8.2) (Table 1). The suicide rate was highest for AI/AN females (12.8), followed by White females (7.6), Asian females (4.2), and persons of more than one race (4.1). The suicide rate for AI/AN females was 3.9 times the rate for females with the lowest rate (i.e., Hispanic females) (3.3).

#### Method and Location of Injury

A firearm was used in more than half (52.5% [7.8 per 100,000 population]) of suicides, followed by hanging, strangulation, or suffocation (24.8% [3.7]) and poisoning (11.3% [1.7]) (Table 1). Among males, the most common method of injury was a firearm (57.8%), followed by hanging, strangulation, or suffocation (24.2%). Among females, a firearm (32.7%) was also the most common method of injury, followed by poisoning (27.8%) and hanging, strangulation, or suffocation (26.9%). Among all suicide decedents, the most common location of suicide was a house or apartment (71.5%), followed by a motor vehicle (5.3%), a natural area (4.2%), a street or highway (2.7%), and a hotel or motel (2.3%).

#### Incident Characteristics

Emergency medical services responded to the scene for a large percentage of suicide decedents (64.8%) (Table 1). Suicide decedents were commonly injured at their homes (63.5%). Male and female suicide decedents had similar percentages of suspected alcohol use at the time of their death (15.1% versus 14.4%, respectively). A child was either present or witnessed the incident for 4.5% of suicide decedents. A small proportion of suicide decedents were recently released from an institutional setting (5.8%) or were experiencing housing instability (2.6%) or homelessness (1.5%) at the time of death.

#### Toxicology Results of Decedent

Toxicology tests for blood alcohol concentration (BAC) were conducted for 46.9% of suicide decedents (Table 2). Among those with positive results for alcohol (i.e., ethanol [40.1%]), 64.1% had a BAC ≥0.08 g/dL. The proportion of decedents tested for a substance varied and among those tested, the positive results differed by substance: amphetamines (38.2% tested, of which 16.9% were positive), barbiturates (32.2% tested, of which 1.6% were positive), benzodiazepines (38.2% tested, of which 20.0% were positive), cannabis (commonly referred to as marijuana; 35.6% tested, of which 28.5% were positive), cocaine (36.7% tested, of which 7.5% were positive), and opioids (including illicit and prescription; 39.3% tested, of which 21.1% were positive). Carbon monoxide was tested for a substantially smaller proportion of decedents (3.2%) but was identified in 40.7% of those decedents. Results for other drugs for which <25% of decedents were tested are provided (Table 2).

**TABLE 2:** Number* and percentage of suicide decedents tested for alcohol and drugs and whose results were positive,^†^ by toxicology variable — National Violent Death Reporting System, 50 states^§^ and District of Columbia, 2022

Toxicology variable	Tested	Positive
	No. (%)	No. (%)
Blood alcohol concentration^¶^	21,057 (46.9)	8,446 (40.1)
Blood alcohol <0.08 g/dL		2,389 (28.3)
Blood alcohol ≥0.08 g/dL		5,416 (64.1)
Blood alcohol positive, level unknown		641 (7.6)
Amphetamines	17,174 (38.2)	2,900 (16.9)
Anticonvulsants	9,027 (20.1)	1,725 (19.1)
Antidepressants	11,041 (24.6)	4,052 (36.7)
Antipsychotics	8,199 (18.3)	1,149 (14.0)
Barbiturates	14,467 (32.2)	238 (1.6)
Benzodiazepines	17,159 (38.2)	3,439 (20.0)
Cannabis**	15,999 (35.6)	4,555 (28.5)
Carbon monoxide	1,438 (3.2)	585 (40.7)
Cocaine	16,497 (36.7)	1,237 (7.5)
Muscle relaxants	9,026 (20.1)	539 (6.0)
Opioids	17,638 (39.3)	3,719 (21.1)
Other drugs or substances^††^	3,151 (7.0)	2,712 (86.1)

* Number of suicide decedents = 44,917. More than one substance could have been tested for or positive per decedent.

^†^ Percentage is of decedents tested for toxicology. Denominator for the percentage positive is the number of decedents tested.

^§^ Data for California are for deaths that occurred in 32 counties, data for Florida are for deaths that occurred in 32 counties, and data for Texas are for deaths that occurred in 13 counties (Supplementary Box).

^¶^ Blood alcohol concentration of ≥0.08 g/dL is over the legal limit in all states and is used as the standard for intoxication.

** Commonly referred to as marijuana.

^††^ Other drugs or substances indicated if any results were positive.

#### Precipitating Circumstances

Precipitating circumstances from coroner or medical examiner records and law enforcement reports were identified in 37,491 (83.5%) suicides (Table 3). Among decedents who had known circumstances, a mental health problem was the most common circumstance identified, with approximately half (48.9%) of decedents having a current diagnosed mental health problem and 27.1% experiencing a depressed mood at the time of death. Among the 18,318 decedents with a current diagnosed mental health problem, depression or dysthymia (71.7%), anxiety disorder (24.0%), and bipolar disorder (14.2%) were the most common diagnoses. Alcohol use problems were reported for 17.3% of suicide decedents, and other substance use problems (unrelated to alcohol) were reported for 16.7% of suicide decedents. Among suicide decedents, 21.4% were receiving mental health or substance use treatment at the time of death and 30.2% had a history of having been treated for a mental health or substance use problem.

**TABLE 3:** Number* and percentage^†^ of suicides, by decedent’s sex and precipitating circumstances — National Violent Death Reporting System, 50 states^§^ and District of Columbia, 2022

Precipitating circumstance	Male	Female	Total
	No. (%)	No. (%)	No. (%)
**Mental health and substance use**			
Current diagnosed mental health problem^¶^	13,167 (44.6)	5,147 (64.6)	18,318 (48.9)
Depression or dysthymia	9,268 (70.4)	3,872 (75.2)	13,143 (71.7)
Anxiety disorder	2,867 (21.8)	1,529 (29.7)	4,399 (24.0)
Bipolar disorder	1,654 (12.6)	937 (18.2)	2,592 (14.2)
Schizophrenia	1,007 (7.6)	272 (5.3)	1,279 (7.0)
Posttraumatic stress disorder	877 (6.7)	253 (4.9)	1,130 (6.2)
Attention deficit disorder or attention hyperactivity disorder	496 (3.8)	110 (2.1)	606 (3.3)
Dementia	252 (1.9)	43 (<1.0)	295 (1.6)
Autism spectrum disorder	143 (1.1)	22 (<1.0)	165 (<1.0)
Obsessive compulsive disorder	76 (<1.0)	25 (<1.0)	101 (<1.0)
Eating disorder	13 (<1.0)	40 (<1.0)	53 (<1.0)
Fetal alcohol syndrome	2 (<1.0)	2 (<1.0)	4 (<1.0)
Other	612 (4.6)	217 (4.2)	829 (4.5)
Unknown	1,038 (7.9)	379 (7.4)	1,417 (7.7)
History of ever being treated for a mental health or substance use problem	7,872 (26.7)	3,441 (43.2)	11,317 (30.2)
Current depressed mood	7,964 (27.0)	2,205 (27.7)	10,170 (27.1)
Current mental health or substance use treatment	5,427 (18.4)	2,604 (32.7)	8,035 (21.4)
Alcohol problem	5,291 (17.9)	1,197 (15.0)	6,488 (17.3)
Substance use problem (excludes alcohol)	4,795 (16.2)	1,475 (18.5)	6,270 (16.7)
Non-adherence to mental health or substance use treatment	1,217 (4.1)	420 (5.3)	1,638 (4.4)
Other addiction (e.g., gambling or sex)	208 (<1.0)	36 (<1.0)	244 (<1.0)
**Interpersonal factor**			
Intimate partner problem	7,137 (24.2)	1,666 (20.9)	8,804 (23.5)
Family relationship problem	1,753 (5.9)	631 (7.9)	2,385 (6.4)
Other death (not suicide) of family member or friend	1,722 (5.8)	517 (6.5)	2,239 (6.0)
Suicide of family member or friend	554 (1.9)	210 (2.6)	764 (2.0)
Perpetrator of interpersonal violence during past month	687 (2.3)	73 (<1.0)	760 (2.0)
Other relationship problem (non-intimate and non-family)	558 (1.9)	160 (2.0)	718 (1.9)
Victim of interpersonal violence during past month	59 (<1.0)	61 (<1.0)	120 (<1.0)
**Life stressor**			
Crisis during previous or upcoming 2 weeks	8,706 (29.5)	1,989 (24.9)	10,696 (28.5)
Physical health problem	5,788 (19.6)	1,374 (17.2)	7,162 (19.1)
Argument or conflict	4,354 (14.8)	1,144 (14.3)	5,499 (14.7)
Victim known to authorities	4,114 (13.9)	936 (11.7)	5,050 (13.5)
Job problem	2,322 (7.9)	391 (4.9)	2,713 (7.2)
Contributing recent criminal legal problem	2,088 (7.1)	218 (2.7)	2,306 (6.2)
Financial problem	1,866 (6.3)	391 (4.9)	2,257 (6.0)
Family stressor	843 (2.9)	326 (4.1)	1,169 (3.1)
Eviction or loss of home	823 (2.8)	212 (2.7)	1,035 (2.8)
Non-criminal legal problem	821 (2.8)	210 (2.6)	1,031 (2.7)
Household known to local authorities	723 (2.4)	258 (3.2)	981 (2.6)
Exposure to disaster	467 (1.6)	118 (1.5)	585 (1.6)
History of traumatic brain injury	370 (1.3)	63 (<1.0)	433 (1.2)
School problem	294 (<1.0)	102 (1.3)	396 (1.1)
History of child abuse or neglect	247 (<1.0)	145 (1.8)	392 (1.0)
Physical fight (two persons, not a brawl)	293 (<1.0)	48 (<1.0)	341 (<1.0)
Living transition or loss of independent living	250 (<1.0)	53 (<1.0)	303 (<1.0)
Traumatic anniversary	211 (<1.0)	87 (1.1)	298 (<1.0)
Caregiver burden	172 (<1.0)	45 (<1.0)	217 (<1.0)
Caretaker abuse or neglect	44 (<1.0)	31 (<1.0)	75 (<1.0)
**Crime and criminal activity**			
Precipitated by another crime	1,156 (3.9)	94 (1.2)	1,250 (3.3)
Crime in progress**	379 (32.8)	21 (22.3)	400 (32.0)
**Suicide and self-harm events**			
History of suicidal thoughts or plans	9,610 (32.6)	3,053 (38.3)	12,665 (33.8)
Left a suicide note	7,911 (26.8)	2,762 (34.6)	10,675 (28.5)
History of attempting suicide	4,309 (14.6)	2,373 (29.8)	6,684 (17.8)
History of non-suicidal self-harm	618 (2.1)	536 (6.7)	1,154 (3.1)
**Suicide disclosure**			
Disclosed suicidal intent	5,864 (19.9)	1,583 (19.9)	7,448 (19.9)
Disclosed intent to whom^††^			
Former or current intimate partner	2,343 (40.0)	521 (32.9)	2,865 (38.5)
Family member (excludes intimate partner)	2,103 (35.9)	619 (39.1)	2,723 (36.6)
Friend or colleague	758 (12.9)	264 (16.7)	1,022 (13.7)
Health care worker	287 (4.9)	129 (8.1)	417 (5.6)
Through social media or other electronic means	286 (4.9)	73 (4.6)	359 (4.8)
Neighbor	85 (1.4)	25 (1.6)	110 (1.5)
Other	585 (10.0)	108 (6.8)	693 (9.3)
Unknown	448 (7.6)	116 (7.3)	564 (7.6)
**Child decedent incident^§§^**			
Previous Child Protective Services report on child victim’s household	14 (1.7)	8 (2.2)	22 (1.9)
Substance use problems in child victim’s household	3 (<1.0)	3 (<1.0)	6 (<1.0)
**Total^¶¶^**	**29,514 (83.0)**	**7,973 (85.3)**	**37,491 (83.5)**

* Includes suicides with one or more precipitating circumstances. More than one circumstance could have been present per decedent.

^†^ Denominator includes those suicides with one or more precipitating circumstances. The sums of percentages in columns exceed 100% because more than one circumstance could have been present per decedent.

^§^ Data for California are for deaths that occurred in 32 counties, data for Florida are for deaths that occurred in 32 counties, and data for Texas are for deaths that occurred in 13 counties (Supplementary Box).

^¶^ Includes decedents with one or more diagnosed current mental health problems; therefore, sums of percentages for the diagnosed conditions exceed 100%. Denominators for the diagnosed conditions include the number of decedents with one or more current diagnosed mental health problems.

** Denominator includes those decedents involved in an incident that was precipitated by another crime.

^††^ Denominator includes decedents who disclosed intent. The sum of percentages exceeds 100% because more than one response could have been present per decedent.

^§§^ Circumstance variables in this category are applicable exclusively to children. Denominator includes decedents aged ≤17 years when circumstances are known (n = 1,181; 812 males, 367 females, and two unknown). Circumstances were unknown for 248 child decedents; total number of child suicide decedents = 1,429.

^¶¶^ Circumstances were unknown for 7,426 decedents (6,056 males, 1,369 females, and one unknown); total number of suicide decedents = 44,917 (35,570 males, 9,342 females, and five unknown).

The most commonly reported interpersonal or life stressor–related precipitating circumstances for suicide were a recent or impending crisis during the previous or upcoming 2 weeks (28.5%) (Supplementary Table 4), an intimate partner problem (23.5%), a physical health problem (19.1%), an argument or conflict (14.7%), and the decedent was known to authorities (13.5%) (Table 3). Among other circumstances related to suicide, 33.8% of decedents had a history of suicidal thoughts or plans, 28.5% left a suicide note, 19.9% had disclosed suicidal intent to another person, and 17.8% had a history of attempting suicide. Among those who disclosed intent, the greatest proportion of disclosures were to a former or current intimate partner (38.5%), followed by a family member other than an intimate partner (36.6%) and a friend or colleague (13.7%).

When examining known circumstances by sex, a larger percentage of female decedents (64.6%) had a current diagnosed mental health problem than did male decedents (44.6%) (Table 3). Female and male suicide decedents had similar percentages of depressed mood at the time of their death (27.7% and 27.0%, respectively). A larger percentage of female decedents (32.7%) than male decedents (18.4%) were known to have been receiving mental health or substance use treatment at the time of death. Suicide events, including leaving a suicide note, history of suicidal thoughts or plans, history of attempting suicide, and history of non-suicidal self-harm, occurred more frequently among female decedents than male decedents.

Known circumstances were identified in 1,181 (82.6%) suicides of children aged <18 years (Table 3). Both male and female child suicide decedents had similar percentages of the two circumstances that only applied to this age group: previous Child Protective Services involvement (1.7% and 2.2%, respectively) and substance use problems in their household (both <1.0%). 

### Homicides

#### Sex, Age Group, and Race and Ethnicity

For 2022, all 50 states (47 states collecting statewide data, 32 California counties, 32 Florida counties, and 13 Texas counties) and the District of Columbia collected NVDRS data on 21,240 incidents involving 22,395 homicide deaths (Supplementary Table 1). The overall homicide rate was 7.4 per 100,000 population (Table 4).

**TABLE 4:** Number, percentage,* and rate^†^ of homicides, by decedent’s selected demographics,^§^ method of injury, location of injury, incident characteristics, and victim-suspect relationship^¶^ — National Violent Death Reporting System, 50 states** and District of Columbia, 2022

Characteristic	Male		Female		Total	
	No. (%)	Rate	No. (%)	Rate	No. (%)	Rate
**Age group, yrs**						
<1	139 (<1.0)	8.2	88 (2.0)	5.4	228 (1.0)	6.8
1–4	186 (1.0)	2.7	128 (2.9)	2.0	314 (1.4)	2.3
5–9	89 (<1.0)	1.0	68 (1.5)	0.8	157 (<1.0)	0.9
10–14	214 (1.2)	2.2	92 (2.1)	1.0	306 (1.4)	1.6
15–19	2,165 (12.0)	21.6	331 (7.5)	3.5	2,496 (11.1)	12.8
20–24	2,645 (14.7)	25.2	504 (11.4)	5.0	3,149 (14.1)	15.3
25–29	2,549 (14.2)	24.7	478 (10.8)	4.8	3,027 (13.5)	15.0
30–34	2,528 (14.1)	23.5	520 (11.8)	5.0	3,048 (13.6)	14.4
35–44	3,473 (19.3)	17.3	782 (17.7)	4.0	4,255 (19.0)	10.7
45–54	1,944 (10.8)	10.5	506 (11.5)	2.8	2,451 (10.9)	6.7
55–64	1,252 (7.0)	6.6	420 (9.5)	2.2	1,672 (7.5)	4.4
65–74	541 (3.0)	3.7	266 (6.0)	1.6	807 (3.6)	2.6
75–84	204 (1.1)	2.9	156 (3.5)	1.8	360 (1.6)	2.3
≥85	44 (<1.0)	2.1	74 (1.7)	1.9	118 (<1.0)	2.0
Unknown	5 (<1.0)	—^††^	1 (<1.0)	—	7 (<1.0)	—
**Race and ethnicity^§§^**						
American Indian or Alaska Native	256 (1.4)	22.6	78 (1.8)	6.7	334 (1.5)	14.6
Asian	167 (<1.0)	1.9	96 (2.2)	1.0	263 (1.2)	1.4
Black or African American	10,584 (58.9)	56.0	1,746 (39.6)	8.5	12,331 (55.1)	31.3
Native Hawaiian or other Pacific Islander	40 (<1.0)	13.8	7 (<1.0)	—	47 (<1.0)	8.2
White	3,506 (19.5)	3.9	1,689 (38.3)	1.8	5,195 (23.2)	2.9
More than one race	216 (1.2)	5.9	86 (1.9)	2.3	302 (1.3)	4.1
Hispanic or Latino	3,120 (17.4)	11.7	692 (15.7)	2.7	3,812 (17.0)	7.3
Unspecified or unknown	89 (<1.0)	—	20 (<1.0)	—	111 (<1.0)	—
**Method of injury**						
Firearm	14,256 (79.3)	9.5	2,820 (63.9)	1.8	17,076 (76.2)	5.6
Sharp instrument	1,337 (7.4)	0.9	513 (11.6)	0.3	1,850 (8.3)	0.6
Blunt instrument	528 (2.9)	0.4	251 (5.7)	0.2	779 (3.5)	0.3
Personal weapons (e.g., hands, fists, or feet)	425 (2.4)	0.3	150 (3.4)	0.1	575 (2.6)	0.2
Hanging, strangulation, or suffocation	144 (<1.0)	0.1	182 (4.1)	0.1	326 (1.5)	0.1
Motor vehicle (e.g., bus, motorcycle, or other transport vehicle)	116 (<1.0)	<0.1	43 (<1.0)	<0.1	159 (<1.0)	<0.1
Poisoning	68 (<1.0)	<0.1	37 (<1.0)	<0.1	105 (<1.0)	<0.1
Fire or burns	48 (<1.0)	<0.1	37 (<1.0)	<0.1	85 (<1.0)	<0.1
Intentional neglect	36 (<1.0)	<0.1	41 (<1.0)	<0.1	77 (<1.0)	<0.1
Fall	28 (<1.0)	<0.1	21 (<1.0)	<0.1	49 (<1.0)	<0.1
Shaking (e.g., shaken baby syndrome)	28 (<1.0)	<0.1	20 (<1.0)	<0.1	48 (<1.0)	<0.1
Drowning	29 (<1.0)	<0.1	12 (<1.0)	—	41 (<1.0)	<0.1
Other (e.g., Taser, electrocution, or nail gun)	22 (<1.0)	—	11 (<1.0)	—	33 (<1.0)	—
Unknown	913 (5.1)	—	276 (6.3)	—	1,192 (5.3)	—
**Location of injury**						
House or apartment	6,651 (37.0)	4.4	2,732 (61.9)	1.8	9,383 (41.9)	3.1
Street or highway	4,194 (23.3)	2.8	399 (9.0)	0.3	4,594 (20.5)	1.5
Motor vehicle	1,852 (10.3)	1.2	423 (9.6)	0.3	2,275 (10.2)	0.8
Commercial or retail area	1,059 (5.9)	0.7	118 (2.7)	<0.1	1,177 (5.3)	0.4
Parking lot, public garage, or public transport	1,013 (5.6)	0.7	113 (2.6)	<0.1	1,126 (5.0)	0.4
Natural area	301 (1.7)	0.2	81 (1.8)	<0.1	382 (1.7)	0.1
Bar or nightclub	303 (1.7)	0.2	32 (<1.0)	<0.1	335 (1.5)	0.1
Park, playground, or sports or athletic area	273 (1.5)	0.2	29 (<1.0)	<0.1	302 (1.3)	0.1
Hotel or motel	210 (1.2)	0.1	71 (1.6)	<0.1	281 (1.3)	<0.1
Other location^¶¶^	1,009 (5.6)	—	172 (3.9)	—	1,181 (5.3)	—
Unknown	1,113 (6.2)	—	244 (5.5)	—	1,359 (6.1)	—
**Incident characteristic**						
Emergency medical services present	12,783 (71.1)	8.5	2,870 (65.0)	1.9	15,653 (69.9)	5.2
Injured at victim’s home	3,227 (17.9)	2.2	1,986 (45.0)	1.3	5,213 (23.3)	1.7
Child present or witnessed incident	1,350 (7.5)	0.9	626 (14.2)	0.4	1,976 (8.8)	0.7
Victim was suspected of alcohol use preceding the incident	1,238 (6.9)	0.8	255 (5.8)	0.2	1,493 (6.7)	0.5
Victim was experiencing homelessness	596 (3.3)	0.4	99 (2.2)	<0.1	695 (3.1)	0.2
Victim was injured at work or while working	388 (2.2)	0.3	83 (1.9)	<0.1	471 (2.1)	0.2
Victim was experiencing housing instability	250 (1.4)	0.2	82 (1.9)	<0.1	332 (1.5)	0.1
Victim was recently released from an institutional setting	256 (1.4)	0.2	64 (1.4)	<0.1	321 (1.4)	0.1
Victim was in public custody when injury occurred	277 (1.5)	0.2	17 (<1.0)	—	294 (1.3)	0.1
Relationship of victim to suspect***						
Acquaintance or friend	1,371 (27.0)	0.9	209 (9.0)	0.1	1,580 (21.4)	0.5
Spouse or intimate partner (current or former)	397 (7.8)	0.3	1,178 (50.8)	0.8	1,575 (21.3)	0.5
Other person, known to victim	1,185 (23.4)	0.8	206 (8.9)	0.1	1,391 (18.8)	0.5
Stranger	906 (17.9)	0.6	176 (7.6)	0.1	1,082 (14.6)	0.4
Other relative	412 (8.1)	0.3	141 (6.1)	<0.1	553 (7.5)	0.2
Child^†††^	283 (5.6)	0.2	187 (8.1)	0.1	470 (6.4)	0.2
Parent^†††^	259 (5.1)	0.2	190 (8.2)	0.1	449 (6.1)	0.2
Rival gang member	96 (1.9)	<0.1	2 (<1.0)	—	98 (1.3)	<0.1
Child of suspect’s boyfriend or girlfriend (e.g., child killed by mother’s boyfriend)	58 (1.1)	<0.1	27 (1.2)	<0.1	85 (1.2)	<0.1
Other relationship^§§§^	102 (2.0)	—	5 (<1.0)	—	107 (1.4)	—
**Total**	**17,978 (100)**	**12.0**	**4,414 (100)**	**2.9**	**22,395 (100)**	**7.4**

* Percentages might not total 100% due to rounding.

^†^ Per 100,000 population.

^§^ Sex was unknown for three decedents.

^¶^ The following sentence can be used as a guide for interpreting victim-suspect relationship: “The victim is the ____________ of the suspect”. For example, when a parent kills a child, the relationship is “Child” not “Parent” (“The victim is the child of the suspect”). Note that this sentence is intended to be a general guide. However, certain relationships might not be captured by this sentence (e.g., other person known to victim; victim was law enforcement officer killed in the line of duty).

** Data for California are for deaths that occurred in 32 counties, data for Florida are for deaths that occurred in 32 counties, and data for Texas are for deaths that occurred in 13 counties (Supplementary Box). Denominators for the rates for California, Florida, and Texas represent only the populations of the counties from which the data were collected.

^††^ Dashes indicate cell data are suppressed because number of decedents is <20 or characteristic response is “Other” or “Unknown.”

^§§^ Persons of Hispanic or Latino (Hispanic) origin might be of any race but are categorized as Hispanic; all racial groups are non-Hispanic.

^¶¶ ^Other location includes (in descending order): jail or prison; supervised residential facility; abandoned house, building, or warehouse; hospital or medical facility; preschool, school, college, or school bus; industrial or construction area; office building; synagogue, church, or temple; farm; railroad tracks; cemetery, graveyard, or other burial ground; bridge; and other unspecified location.

*** Percentage is based on the number of homicide decedents with a known victim-to-suspect relationship (n = 7,390 [33.0%]; 5,069 [28.2%] males and 2,321 [52.6%] females); victim-suspect relationship was unknown for 15,005 decedents.

^††† ^Includes adoptive family members (e.g., adopted child), stepfamily members (e.g., stepparent), and foster family members (e.g., foster child).

^§§§^ Other victim-suspect relationship includes (in descending order): intimate partner of suspect’s parent (e.g., teenager kills his mother’s boyfriend), victim was law enforcement officer injured in the line of duty, and victim was injured by a law enforcement officer.

The overall homicide rate for males (12.0 per 100,000 population) was 4.1 times the rate for females (2.9), and the homicide rates were higher for males than for females across all age groups (Table 4). The homicide rate was highest among adults aged 20–24 years (15.3). The homicide rate for males aged 20–24 years (25.2) was approximately five times the rate for females in the same age group (5.0). For males, the rate of homicide was highest among adults aged 20–24 years (25.2) and 25–29 years (24.7). For females, the rate of homicide was highest among infants (i.e., aged <1 year) (5.4) and adults aged 20–24 years and 30–34 years (both 5.0). Among all children who were homicide victims, the homicide rate for infants (6.8) was 3.0 times the overall rate for children aged 1–4 years (2.3) and 7.6 times the rate for children aged 5–9 years (0.9).

Black persons accounted for 58.9% of male homicide victims and 39.6% of female homicide victims (Table 4). Black males had the highest rate of homicide compared with males in all other racial and ethnic groups (56.0 per 100,000 population); this rate was 29.5 times the rate for Asian males (1.9), 14.4 times the rate for White males (3.9), 9.5 times the rate for males of more than one race (5.9), 4.8 times the rate for Hispanic males (11.7), 4.1 times the rate for NH/PI males (13.8), and 2.5 times the rate for AI/AN males (22.6). Among females, the homicide rate was also highest among Black females (8.5), followed by AI/AN females (6.7), Hispanic females (2.7), females of more than one race (2.3), White females (1.8), and Asian females (1.0).

#### Method and Location of Injury

Firearms were used in 76.2% of homicides, making them the most commonly used weapon, followed by a sharp instrument (8.3%); a blunt instrument (3.5%); personal weapons (e.g., hands, feet, or fists; 2.6%); and hanging, strangulation, or suffocation (1.5%) (Table 4). The method was unknown in 5.3% of homicides. A firearm was the most common method of injury for both males (79.3%) and females (63.9%); however, the firearm homicide rate for males (9.5 per 100,000 population) was 5.3 times the rate for females (1.8). For males, the next most common methods of injury apart from firearms were sharp instruments (7.4%), blunt instruments (2.9%), and personal weapons (2.4%). For females, sharp instruments (11.6%); blunt instruments (5.7%); and hanging, strangulation, or suffocation (4.1%) were additional common methods of injury. Among all homicide victims, a house or apartment was the most common location of injury (41.9%); followed by a street or highway (20.5%); a motor vehicle (10.2%); a commercial or retail area (5.3%); and a parking lot, public garage, or public transport (5.0%). A larger proportion of homicides among females (61.9%) than among males (37.0%) occurred at a house or apartment, whereas a larger proportion of homicides among males (23.3%) than among females (9.0%) occurred on a street or highway.

#### Incident Characteristics

Emergency medical services responded to the scene for a large percentage of homicide victims (69.9%) (Table 4). A larger proportion of homicides among females than among males occurred at the victim’s home (45.0% and 17.9%, respectively) and involved a child who was present or witnessed the incident (14.2% and 7.5%, respectively). Among all homicide victims, 6.7% of victims were suspected of alcohol use preceding the incident. A small proportion of all homicide victims were experiencing homelessness (3.1%) or housing instability (1.5%) or were recently injured at work or while working (2.1%).

#### Victim-Suspect Relationship

The relationship of the victim to the suspect was known for 33.0% of homicides (28.2% of males and 52.6% of females) (Table 4). For males, when the relationship was known, the victim-suspect relationship was most often an acquaintance or friend (27.0%), other person known to the victim, but the exact nature of the relationship was unclear (23.4%), a stranger (17.9%), a relative other than a parent or child (8.1%), or a current or former intimate partner (7.8%). For females, when the victim-suspect relationship was known, approximately half (50.8%) were a current or former intimate partner, followed by an acquaintance or friend (9.0%), other person known to victim, but the exact nature of the relationship was unclear (8.9%), a parent (8.2%), a child (8.1%), or a stranger (7.6%).

#### Precipitating Circumstances

Precipitating circumstances were identified in 70.6% of homicides (Table 5). One third of homicides with known circumstances were precipitated by an argument or conflict (34.9%), and 15.0% of homicides with known circumstances were related to intimate partner violence. Intimate partner violence–related deaths include deaths related to conflict or violence between current or former intimate partners and also deaths associated with intimate partner violence that are not deaths of the intimate partners themselves (e.g., a former boyfriend killing an ex-partner’s new boyfriend). Other common precipitating circumstances included the victim being known to authorities (13.4%), a physical fight between two persons (13.2%), a drive-by shooting (11.8%), and drug involvement (e.g., relating to drug use or illegal drug trafficking [8.3%]). Homicides also were commonly precipitated by another crime (22.1%); in 68.5% of those cases, the crime was in progress at the time of the incident. The most frequent types of precipitating crimes were assault or homicide (51.3%), robbery (25.3%), burglary (10.8%), drug trade** (10.6%), motor vehicle theft (4.8%), rape or sexual assault (1.9%), and arson (1.1%) (Supplementary Table 5). A recent or impending crisis during the previous or upcoming 2 weeks was reported for 7.5% of decedents (Supplementary Table 6). Toxicology results for homicide deaths are available (Supplementary Table 7).

**TABLE 5:** Number* and percentage^† ^of homicides, by decedent’s sex and precipitating circumstances — National Violent Death Reporting System, 50 states^§ ^and District of Columbia, 2022

Precipitating circumstance	Male	Female	Total
	No. (%)	No. (%)	No. (%)
**Mental health and substance use**			
Substance use problem (excludes alcohol)	1,609 (12.9)	357 (10.8)	1,966 (12.4)
Current diagnosed mental health problem	637 (5.1)	294 (8.9)	931 (5.9)
Alcohol problem	499 (4.0)	117 (3.5)	616 (3.9)
History of ever being treated for a mental health or substance use problem	294 (2.3)	151 (4.6)	445 (2.8)
Current mental health or substance use treatment	156 (1.2)	99 (3.0)	255 (1.6)
Non-adherence to mental health or substance use treatment	32 (<1.0)	11 (<1.0)	43 (<1.0)
Current depressed mood	22 (<1.0)	20 (<1.0)	42 (<1.0)
Other addiction (e.g., gambling or sex)	21 (<1.0)	6 (<1.0)	27 (<1.0)
**Interpersonal factor**			
Intimate partner violence–related	998 (8.0)	1,382 (41.8)	2,380 (15.0)
Other relationship problem (non-intimate and non-family)	1,006 (8.0)	152 (4.6)	1,158 (7.3)
Family relationship problem	608 (4.9)	260 (7.9)	868 (5.5)
Jealousy (lovers’ triangle)	257 (2.1)	89 (2.7)	346 (2.2)
Victim of interpersonal violence during past month	91 (<1.0)	127 (3.8)	218 (1.4)
Perpetrator of interpersonal violence during past month	165 (1.3)	22 (<1.0)	187 (1.2)
**Life stressor**			
Argument or conflict	4,511 (36.1)	1,001 (30.3)	5,512 (34.9)
Victim known to authorities	1,764 (14.1)	359 (10.9)	2,123 (13.4)
Physical fight (two persons, not a brawl)	1,805 (14.4)	285 (8.6)	2,090 (13.2)
Crisis during previous or upcoming 2 weeks	886 (7.1)	294 (8.9)	1,180 (7.5)
Household had contact with local authorities	280 (2.2)	261 (7.9)	541 (3.4)
Family stressor	149 (1.2)	84 (2.5)	233 (1.5)
History of child abuse or neglect	102 (<1.0)	70 (2.1)	172 (1.1)
Exposure to disaster	30 (<1.0)	11 (<1.0)	41 (<1.0)
Living transition or loss of independent living	12 (<1.0)	10 (<1.0)	22 (<1.0)
**Crime and criminal activity**			
Precipitated by another crime	2,913 (23.3)	575 (17.4)	3,488 (22.1)
Crime in progress^¶^	2,002 (68.7)	388 (67.5)	2,390 (68.5)
Drug involvement	1,164 (9.3)	142 (4.3)	1,306 (8.3)
Gang-related	740 (5.9)	74 (2.2)	814 (5.1)
**Homicide event**			
Drive-by shooting	1,642 (13.1)	228 (6.9)	1,870 (11.8)
Victim used a weapon	1,194 (9.5)	69 (2.1)	1,263 (8.0)
Walk-by assault	1,055 (8.4)	141 (4.3)	1,196 (7.6)
Caretaker abuse or neglect led to death	424 (3.4)	405 (12.3)	829 (5.2)
Justifiable self-defense	496 (4.0)	18 (<1.0)	514 (3.3)
Mentally ill suspect**	227 (2.0)	181 (5.6)	408 (2.8)
Random violence	289 (2.3)	87 (2.6)	376 (2.4)
Victim was a bystander	214 (1.7)	149 (4.5)	363 (2.3)
Brawl	309 (2.5)	36 (1.1)	345 (2.2)
Victim was an intervener assisting a crime victim	144 (1.2)	19 (<1.0)	163 (1.0)
Stalking	33 (<1.0)	34 (1.0)	67 (<1.0)
Victim was a police officer on duty	52 (<1.0)	2 (<1.0)	54 (<1.0)
Prostitution	26 (<1.0)	26 (<1.0)	52 (<1.0)
Hate crime	15 (<1.0)	11 (<1.0)	26 (<1.0)
Mercy killing	1 (<1.0)	11 (<1.0)	12 (<1.0)
Terrorist attack	5 (<1.0)	6 (<1.0)	11 (<1.0)
**Child victim incident^††^**			
Previous Child Protective Services report on victim’s household	73 (6.4)	38 (9.0)	111 (7.1)
Substance use in victim’s household	42 (3.7)	22 (5.2)	64 (4.1)
Corporal punishment	10 (<1.0)	8 (1.9)	18 (1.2)
**Total^§§^**	**12,511 (69.6)**	**3,303 (74.8)**	**15,815 (70.6)**

* Includes homicides with one or more precipitating circumstances. Total numbers do not equal the sums of the columns because more than one circumstance could have been present per decedent.

^†^ Denominator includes those homicides with one or more precipitating circumstances. The sums of percentages in columns exceed 100% because more than one circumstance could have been present per decedent.

^§^ Data for California are for deaths that occurred in 32 counties, data for Florida are for deaths that occurred in 32 counties, and data for Texas are for deaths that occurred in 13 counties (Supplementary Box).

^¶^ Denominator includes those decedents involved in an incident that was precipitated by another crime.

** Denominator includes those decedents with informative (not missing or unknown) data in one or more of these suspect variables: sex, age, race and ethnicity, or victim-suspect relationship: 14,409 total (11,197 males, 3,211 females, and one unknown).

^††^ Circumstance variables in this category are applicable exclusively to children. Denominator includes decedents aged 0–17 years when circumstances are known (1,141 males, 423 females, and 1,564 total). Circumstances were unknown for 569 decedents aged 0–17 years (458 males, 110 females, and one unknown).

^§§^ Circumstances were unknown for 6,580 decedents (5,467 males, 1,111 females, and two unknown); total number of homicide decedents = 22,395 (17,978 males, 4,414 females, and three unknown).

Among the identified homicide circumstances, multiple differences were noted by decedent’s sex, and intimate partner violence demonstrated the largest percentage difference. Intimate partner violence was a precipitating circumstance for approximately 41.8% of homicides among females but only 8.0% of homicides among males (Table 5). In incidents for which intimate partner violence was a precipitating circumstance and the victim-suspect relationship was known, the suspect was a current or former intimate partner in 90.9% of homicides among females and 45.4% of homicides among males (data not shown). Females were more often the direct victims of intimate partner violence–related homicides, whereas males were more often corollary victims (i.e., persons who were not involved in the intimate partner relationship). A larger proportion of homicides of females than males also resulted from caregiver abuse or neglect (12.3% versus 3.4%) or the victim’s household had contact with local authorities (7.9% versus 2.2%) (Table 5). A larger proportion of homicides of males than of females were precipitated by another crime (23.3% versus 17.4%), precipitated by an argument or conflict (36.1% versus 30.3%), preceded by a physical fight (14.4% versus 8.6%), or involved drugs (9.3% versus 4.3%) or a drive-by shooting (13.1% versus 6.9%). A larger proportion of male homicide victims than female homicide victims were reported to have used a weapon during the incident (9.5% versus 2.1%).

Known circumstances of incidents were identified in 1,564 (73.3%) homicides of children aged <18 years (Table 5). A larger proportion of female than of male victims’ households had previous Child Protective Services reports (9.0% versus 6.4%).

### Legal Intervention Deaths

#### Sex, Age Group, and Race and Ethnicity

For 2022, all 50 states (47 states collecting statewide data, 32 California counties, 32 Florida counties, and 13 Texas counties) and the District of Columbia collected NVDRS data on 1,004 incidents involving 1,014 legal intervention deaths (Supplementary Table 1). Nearly all legal intervention deaths were among males (94.9%). The highest rate of legal intervention death by age group was among males aged 30–34 years (1.6 per 100,000 population), followed by males aged 25–29 years and 35–44 years (both 1.3) (Table 6). Although White males accounted for 45.3% of all male legal intervention deaths, AI/AN males had the highest legal intervention death rate (3.2), representing a rate 6.4 times that for White males (0.5). The legal intervention death rate for Black males (1.4) was 2.8 times the rate for White males. The legal intervention death rate for Hispanic males was 0.7.

**TABLE 6:** Number, percentage,* and rate^†^ of legal intervention^§^ deaths, by decedent’s selected demographics, method of injury, location of injury, and incident characteristics — National Violent Death Reporting System, 50 states^¶^ and District of Columbia, 2022

Characteristic	Male		Female		Total	
	No. (%)	Rate	No. (%)	Rate	No. (%)	Rate
**Age group, yrs**						
<10	0 (—)	—**	0 (—)	—	0 (—)	—
10–14	1 (<1.0)	—	0 (—)	—	1 (<1.0)	—
15–19	46 (4.8)	0.5	0 (—)	—	46 (4.5)	0.2
20–24	95 (9.9)	0.9	6 (11.5)	—	101 (10.0)	0.5
25–29	134 (13.9)	1.3	5 (9.6)	—	139 (13.7)	0.7
30–34	170 (17.7)	1.6	4 (7.7)	—	174 (17.2)	0.8
35–44	259 (26.9)	1.3	20 (38.5)	0.1	279 (27.5)	0.7
45–54	141 (14.7)	0.8	10 (19.2)	—	151 (14.9)	0.4
55–64	80 (8.3)	0.4	4 (7.7)	—	84 (8.3)	0.2
65–74	28 (2.9)	0.2	2 (3.8)	—	30 (3.0)	0.1
75–84	7 (<1.0)	—	1 (1.9)	—	8 (<1.0)	—
≥85	1 (<1.0)	—	0 (—)	—	1 (<1.0)	—
**Race and ethnicity^††^**						
American Indian or Alaska Native	36 (3.7)	3.2	0 (—)	—	36 (3.6)	1.6
Asian	13 (1.4)	—	2 (3.8)	—	15 (1.5)	—
Black or African American	264 (27.4)	1.4	19 (36.5)	—	283 (27.9)	0.7
Native Hawaiian or other Pacific Islander	3 (<1.0)	—	0 (—)	—	3 (<1.0)	—
White	436 (45.3)	0.5	25 (48.1)	<0.1	461 (45.5)	0.3
More than one race	8 (<1.0)	—	0 (—)	—	8 (<1.0)	—
Hispanic or Latino	197 (20.5)	0.7	5 (9.6)	—	202 (19.9)	0.4
Unspecified or unknown	5 (<1.0)	—	1 (1.9)	—	6 (<1.0)	—
**Method of injury**						
Firearm	837 (87.0)	0.6	40 (76.9)	<0.1	877 (86.5)	0.3
Motor vehicle (e.g., bus, motorcycle, or other transport vehicles)	44 (4.6)	<0.1	8 (15.4)	—	52 (5.1)	<0.1
Personal weapons (e.g., hands, fists, or feet)	8 (<1.0)	—	1 (1.9)	—	9 (<1.0)	—
Sharp instrument	6 (<1.0)	—	0 (—)	—	6 (<1.0)	—
Blunt instrument	3 (<1.0)	—	1 (1.9)	—	4 (<1.0)	—
Fall	3 (<1.0)	—	1 (1.9)	—	4 (<1.0)	—
Poisoning	3 (<1.0)	—	0 (—)	—	3 (<1.0)	—
Drowning	2 (<1.0)	—	0 (—)	—	2 (<1.0)	—
Fire or burns	2 (<1.0)	—	0 (—)	—	2 (<1.0)	—
Hanging, strangulation, or suffocation	0 (—)	—	1 (1.9)	—	1 (<1.0)	—
Other (e.g., Taser, electrocution, or nail gun)	11 (1.1)	—	0 (—)	—	11 (1.1)	—
Unknown	43 (4.5)	—	0 (—)	—	43 (4.2)	—
**Location of injury**						
House or apartment	330 (34.3)	0.2	24 (46.2)	<0.1	354 (34.9)	0.1
Street or highway	243 (25.3)	0.2	10 (19.2)	—	253 (25.0)	<0.1
Motor vehicle	105 (10.9)	<0.1	8 (15.4)	—	113 (11.1)	<0.1
Commercial or retail area	57 (5.9)	<0.1	4 (7.7)	—	61 (6.0)	<0.1
Parking lot, public garage, or public transport	56 (5.8)	<0.1	2 (3.8)	—	58 (5.7)	<0.1
Natural area	27 (2.8)	<0.1	0 (—)	—	27 (2.7)	<0.1
Other location^§§^	83 (8.6)	—	2 (3.8)	—	85 (8.4)	—
Unknown	61 (6.3)	—	2 (3.8)	—	63 (6.2)	—
**Incident characteristic**						
Emergency medical services present	766 (79.6)	0.5	43 (82.7)	<0.1	809 (79.8)	0.3
Victim was in public custody when injury occurred	332 (34.5)	0.2	16 (30.8)	—	348 (34.3)	0.1
Injured at victim’s home	217 (22.6)	0.1	15 (28.8)	—	232 (22.9)	<0.1
Victim was suspected of alcohol use preceding the incident	113 (11.7)	<0.1	4 (7.7)	—	117 (11.5)	<0.1
Child present or witnessed incident	62 (6.4)	<0.1	5 (9.6)	—	67 (6.6)	<0.1
Victim was recently released from an institutional setting	37 (3.8)	<0.1	3 (5.8)	—	40 (3.9)	<0.1
Victim was experiencing housing instability	29 (3.0)	<0.1	3 (5.8)	—	32 (3.2)	<0.1
Victim was experiencing homelessness	24 (2.5)	<0.1	3 (5.8)	—	27 (2.7)	<0.1
Victim was injured at work or while working	6 (<1.0)	—	0 (—)	—	6 (<1.0)	—
**Total**	**962 (100)**	**0.6**	**52 (100)**	**<0.1**	**1,014 (100)**	**0.3**

* Percentages might not total 100% due to rounding.

^†^ Per 100,000 population.

^§^ The term “legal intervention” does not denote the lawfulness or legality of the circumstances surrounding the death.

^¶^ Data for California are for deaths that occurred in 32 counties, data for Florida are for deaths that occurred in 32 counties, and data for Texas are for deaths that occurred in 13 counties (Supplementary Box). Denominators for the rates for California, Florida, and Texas represent only the populations of the counties from which the data were collected.

** Dashes indicate cell data are suppressed because number of decedents is <20 or characteristic response is “Other” or “Unknown.”

^††^ Persons of Hispanic or Latino (Hispanic) origin might be of any race but are categorized as Hispanic; all racial groups are non-Hispanic.

^§§^ Other location includes (in descending order): hotel or motel; park, playground, or sports or athletic area; office building; jail or prison; preschool, school, college, or school bus; hospital or medical facility; synagogue, church, or temple; supervised residential facility; railroad tracks; bar or nightclub; industrial or construction area; bridge; cemetery, graveyard, or other burial ground; and other unspecified location.

#### Method and Location of Injury

A firearm was used in a majority (86.5%) of legal intervention deaths (Table 6). Legal intervention deaths occurred most frequently in a house or apartment (34.9%), followed by a street or highway (25.0%) or a motor vehicle (11.1%).

#### Incident Characteristics

Among all legal intervention deaths, emergency medical services responded to the scene for a large percentage of deaths (79.8%), and approximately one third of decedents (34.3%) were in public custody when the injury occurred (Table 6). Approximately one quarter of legal intervention deaths occurred at the decedent’s home (22.9%), 11.5% of decedents were suspected of alcohol use preceding the incident, and, during 6.6% of the deaths, a child was present or witnessed the incident. A small proportion of legal intervention deaths involved decedents experiencing housing instability (3.2%) or homelessness (2.7%) at the time of death.

#### Precipitating Circumstances

Precipitating circumstances were identified in 94.0% of legal intervention deaths (Table 7). Among legal intervention deaths with known circumstances, the decedent reportedly used a weapon in 68.8% of legal intervention death cases. The decedent was known to authorities before the fatal incident in 34.6% of legal intervention deaths. In 23.0% of legal intervention deaths, a substance use problem (other than alcohol) was reported as a contributing factor, and 19.3% of decedents reportedly had a current diagnosed mental health problem. An argument or conflict precipitated more legal intervention deaths than a physical fight (12.6% and 6.9%, respectively). A recent or impending crisis during the previous or upcoming 2 weeks was reported in 11.6% of legal intervention deaths (Supplementary Table 8). Having ever been treated for a mental health or substance use problem (10.9%), being a perpetrator of interpersonal violence during the past month (8.8%), having an alcohol problem (8.0%), and family relationship problems (6.1%) were other precipitating circumstances. Toxicology results for legal intervention deaths are available (Supplementary Table 9).

**TABLE 7:** Number* and percentage^†^ of legal intervention^§^ deaths, by decedent’s sex and precipitating circumstances — National Violent Death Reporting System, 50 states^¶^ and District of Columbia, 2022

Precipitating circumstance	Male	Female	Total
	No. (%)	No. (%)	No. (%)
**Mental health and substance use**			
Substance use problem (excludes alcohol)	206 (22.8)	13 (25.5)	219 (23.0)
Current diagnosed mental health problem	171 (19.0)	13 (25.5)	184 (19.3)
History of ever being treated for a mental health or substance use problem	94 (10.4)	10 (19.6)	104 (10.9)
Alcohol problem	70 (7.8)	6 (11.8)	76 (8.0)
Current mental health or substance use treatment	47 (5.2)	5 (9.8)	52 (5.5)
Non-adherence to mental health or substance use treatment	35 (3.9)	2 (3.9)	37 (3.9)
Current depressed mood	22 (2.4)	2 (3.9)	24 (2.5)
Other addiction (e.g., gambling or sex)	2 (<1.0)	0 (—)	2 (<1.0)
**Interpersonal factor**			
Perpetrator of interpersonal violence during past month	81 (9.0)	3 (5.9)	84 (8.8)
Family relationship problem	58 (6.4)	0 (—)	58 (6.1)
Intimate partner violence–related	55 (6.1)	1 (2.0)	56 (5.9)
Other relationship problem (non-intimate and non-family)	15 (1.7)	2 (3.9)	17 (1.8)
Victim of interpersonal violence during past month	3 (<1.0)	1 (2.0)	4 (<1.0)
Jealousy (lovers’ triangle)	3 (<1.0)	0 (—)	3 (<1.0)
**Life stressor**			
Victim known to authorities	316 (35.0)	14 (27.5)	330 (34.6)
Argument or conflict	113 (12.5)	7 (13.7)	120 (12.6)
Crisis during previous or upcoming 2 weeks	106 (11.8)	5 (9.8)	111 (11.6)
Physical fight (two persons, not a brawl)	61 (6.8)	5 (9.8)	66 (6.9)
Household had contact with local authorities	38 (4.2)	2 (3.9)	40 (4.2)
Family stressor	7 (<1.0)	1 (2.0)	8 (<1.0)
History of child abuse or neglect	4 (<1.0)	0 (—)	4 (<1.0)
Living transition or loss of independent living	2 (<1.0)	0 (—)	2 (<1.0)
Exposure to disaster	1 (<1.0)	0 (—)	1 (<1.0)
**Crime and criminal activity**			
Drug involvement	29 (3.2)	3 (5.9)	32 (3.4)
Gang-related	4 (<1.0)	0 (—)	4 (<1.0)
**Legal intervention event**			
Victim used a weapon	628 (69.6)	28 (54.9)	656 (68.8)
Brawl	13 (1.4)	1 (2.0)	14 (1.5)
Random violence	6 (<1.0)	0 (—)	6 (<1.0)
Stalking	5 (<1.0)	0 (—)	5 (<1.0)
Prostitution	1 (<1.0)	2 (3.9)	3 (<1.0)
Victim was a bystander	1 (<1.0)	0 (—)	1 (<1.0)
Victim was an intervener assisting a crime victim	1 (<1.0)	0 (—)	1 (<1.0)
Caretaker abuse or neglect	1 (<1.0)	0 (—)	1 (<1.0)
Total**	**902 (93.8)**	**51 (98.1)**	**953 (94.0)**

* Includes deaths with one or more precipitating circumstances. Total numbers do not equal the sums of the columns because more than one circumstance could have been present per decedent.

^†^ Denominator includes those deaths with one or more precipitating circumstances. The sums of percentages in columns exceed 100% because more than one circumstance could have been present per decedent.

^§^ The term “legal intervention” does not denote the lawfulness or legality of the circumstances surrounding the death.

^¶^ Data for California are for deaths that occurred in 32 counties, data for Florida are for deaths that occurred in 32 counties, and data for Texas are for deaths that occurred in 13 counties (Supplementary Box).

** Circumstances were unknown for 61 decedents (60 males and one female); total number of legal intervention deaths = 1,014 (962 males and 52 females).

### Unintentional Firearm Injury Deaths

#### Sex, Age Group, and Race and Ethnicity

In 2022, a total of 50 NVDRS states (47 states collecting statewide data, 32 California counties, 32 Florida counties, and 13 Texas counties) and the District of Columbia collected data on 528 incidents involving 530 unintentional firearm injury deaths (Supplementary Table 1). Nearly half (n = 233; 44.0%) of these deaths were self-inflicted, and 200 deaths (37.7%) were known to be inflicted by another person; for the remaining 97 deaths (18.3%), whether the injury was self- or other-inflicted was unknown (data not shown). Males accounted for 87.0% of decedents (Table 8). The largest proportion of decedents were White (46.4%), followed by Black (37.7%). Persons aged ≤24 years accounted for more than half (57.7%) of all unintentional firearm injury deaths.

**TABLE 8:** Number and percentage* of unintentional firearm injury deaths, by decedent’s selected demographics, location of injury, type of firearm, and incident characteristics — National Violent Death Reporting System, 50 states^†^ and District of Columbia, 2022

Characteristic	No. (%)
**Sex**	
Male	461 (87.0)
Female	69 (13.0)
**Race and ethnicity^§^**	
American Indian or Alaska Native	7 (1.3)
Asian	4 (<1.0)
Black or African American	200 (37.7)
Native Hawaiian or other Pacific Islander	2 (<1.0)
White	246 (46.4)
More than one race	6 (1.1)
Hispanic or Latino	63 (11.9)
Unspecified or unknown	2 (<1.0)
**Age group, yrs**	
<1	1 (<1.0)
1–4	59 (11.1)
5–9	19 (3.6)
10–14	45 (8.5)
15–19	112 (21.1)
20–24	70 (13.2)
25–29	40 (7.5)
30–34	32 (6.0)
35–44	42 (7.9)
45–54	31 (5.8)
55–64	34 (6.4)
65–74	27 (5.1)
75–84	13 (2.5)
≥85	5 (<1.0)
**Location of injury**	
House or apartment	415 (78.3)
Motor vehicle	32 (6.0)
Natural area	17 (3.2)
Street or highway	14 (2.6)
Commercial or retail area	8 (1.5)
Parking lot, public garage, or public transport	5 (<1.0)
Park, playground, or sports or athletic area	4 (<1.0)
Hotel or motel	4 (<1.0)
Industrial or construction area	1 (<1.0)
Abandoned house, building, or warehouse	1 (<1.0)
Preschool, school, college, or school bus	1 (<1.0)
Synagogue, church, or temple	1 (<1.0)
Cemetery, graveyard, or other burial ground	1 (<1.0)
Other unspecified location	4 (<1.0)
Unknown	22 (4.2)
**Firearm type**	
Handgun	331 (62.5)
Rifle	44 (8.3)
Shotgun	27 (5.1)
Other	6 (1.1)
Unknown	122 (23.0)
**Incident characteristic**	
Emergency medical services present	389 (73.4)
Injured at victim’s home	278 (52.5)
Child present or witnessed incident	153 (28.9)
Victim was suspected of alcohol use preceding the incident	67 (12.6)
Victim was recently released from an institutional setting	5 (<1.0)
Victim was injured at work or while working	3 (<1.0)
Victim was experiencing homelessness	2 (<1.0)
Victim was experiencing housing instability	2 (<1.0)
**Total**	**530 (100)**

* Percentages might not total 100% due to rounding.

^†^ Data for California are for deaths that occurred in 32 counties, data for Florida are for deaths that occurred in 32 counties, and data for Texas are for deaths that occurred in 13 counties (Supplementary Box).

^§^ Persons of Hispanic or Latino (Hispanic) origin might be of any race but are categorized as Hispanic; all racial groups are non-Hispanic.

#### Location of Injury and Firearm Type

Among unintentional firearm injury deaths, 78.3% occurred in a house or apartment, followed by a motor vehicle (6.0%) or a natural area (3.2%) (Table 8). The majority of unintentional firearm injury deaths involved a handgun (62.5%), followed by a rifle (8.3%) or a shotgun (5.1%). The firearm type was unknown in approximately one quarter (23.0%) of unintentional firearm injury deaths.

#### Incident Characteristics

Emergency medical services responded to the scene for most unintentional firearm injury deaths (73.4%) (Table 8). Approximately half of all unintentional firearm injury deaths occurred at the decedent’s home (52.5%), and a child was present or witnessed the incident in 28.9% of these deaths. Furthermore, 12.6% of the decedents were suspected of alcohol use preceding the incident.

#### Context and Circumstances of Injury

The context and circumstances of injury were identified in 84.0% of unintentional firearm injury deaths (Table 9). Among those with context and circumstance information, the most common context of injury of unintentional firearm injury deaths was playing with a firearm (41.6%). Other contexts of injury were showing the firearm to others (11.2%), cleaning the firearm (8.1%), and loading or unloading the firearm (3.6%). Approximately one fourth (23.4%) of unintentional firearm injury deaths were precipitated by a person unintentionally pulling the trigger; 20.0% resulted from a person mistakenly thinking the firearm was unloaded, with 9.2% a disengaged magazine, and 10.8% for another reason; and 7.9% of deaths were precipitated by the firearm being mistaken for a toy.

**TABLE 9:** Number and percentage* of unintentional firearm injury deaths, by context and circumstances of injury — National Violent Death Reporting System, 50 states^†^ and District of Columbia, 2022

Characteristic	No. (%)
**Context of injury**	
Playing with firearm	185 (41.6)
Showing firearm to others	50 (11.2)
Cleaning firearm	36 (8.1)
Loading or unloading firearm	16 (3.6)
Hunting	11 (2.5)
Target shooting	5 (1.1)
Celebratory firing	1 (<1.0)
Self-defensive shooting	1 (<1.0)
Other context of injury	111 (24.9)
**Circumstances of injury**	
Unintentionally pulled trigger	104 (23.4)
Thought firearm was unloaded (not because magazine disengaged)	48 (10.8)
Thought firearm was unloaded, magazine disengaged	41 (9.2)
Firearm was mistaken for a toy	35 (7.9)
Firearm was dropped	28 (6.3)
Firearm fired due to defect or malfunction	12 (2.7)
Thought firearm safety was engaged	7 (1.6)
Firearm fired while holstering	7 (1.6)
Bullet ricocheted	3 (<1.0)
Firearm fired while handling safety lock	2 (<1.0)
Other mechanism of injury	70 (15.7)
**Child victim incident^§^**	
Substance use in victim’s household	6 (3.6)
Previous Child Protective Services report on victim’s household	3 (1.8)
**Total^¶^**	**445 (84.0)**

* Percentages might exceed 100% because one or more circumstances could have been known per death. Number and percentage are reported when the number of deaths is <5 because no particular circumstance identifies a single death. Denominator includes those deaths with one or more precipitating circumstances.

^†^ Data for California are for deaths that occurred in 32 counties, data for Florida are for deaths that occurred in 32 counties, and data for Texas are for deaths that occurred in 13 counties (Supplementary Box).

^§^ Variables in this category apply exclusively to child victims when circumstances were known. Denominator includes decedents aged 0–17 years, n = 165; circumstances were unknown for 20 child decedents.

^¶^ Circumstances were unknown for 85 decedents; total number of unintentional firearm injury decedents = 530.

Known circumstances of incidents were identified in 165 (89.2%) unintentional firearm injury deaths of children aged <18 years. Substance use problems were reported in 3.6% of child victims’ households and, for 1.8% of child deaths, the victim’s household had previous Child Protective Services involvement. 

### Deaths of Undetermined Intent

#### Sex, Age Group, and Race and Ethnicity

In 2022, all 50 states (47 states collecting statewide data, 32 California counties, 32 Florida counties, and 13 Texas counties) and the District of Columbia collected NVDRS data on 5,252 incidents involving 5,292 deaths of undetermined intent (Supplementary Table 1). The overall rate of deaths of undetermined intent was 1.7 per 100,000 population (Supplementary Table 10). The rate of deaths of undetermined intent was higher among males (2.3) than among females (1.2). More than half (57.4%) of deaths of undetermined intent were among adults aged 35–64 years. The rate of deaths of undetermined intent was highest among male infants (i.e., aged <1 year) (4.5), followed by males aged 35–44 (3.9) and males aged 30–34 years and 45–54 years (both 3.4). The rate of deaths of undetermined intent among all infants was 3.8. Although White persons accounted for the majority (60.1% [1.7]) of deaths of undetermined intent, AI/AN persons had the highest rate (4.0). Among males, AI/AN males (5.4) and Black males (4.8) had the highest rates of deaths of undetermined intent. Among females, AI/AN females had the highest rate of deaths of undetermined intent (2.6), followed by Black females (2.0).

#### Method and Location of Injury

Poisoning was the most common method of injury in deaths of undetermined intent (60.7%) (see Toxicology Results of Decedent and Supplementary Table 11), followed by a firearm (5.1%); drowning (4.8%); a blunt instrument (4.2%); fire or burns (3.2%); a fall (2.8%); a motor vehicle (2.8%); and hanging, strangulation, or suffocation (2.4%) (Supplementary Table 10). Sharp instruments, personal weapons, intentional neglect, shaking, and other methods were each used as method of injury in <1.0% of undetermined intent deaths; the method of injury was unknown for 11.2%. The majority of deaths of undetermined intent occurred in a house or apartment (60.3%), followed by a natural area (5.7%), a street or highway (4.5%), or a hotel or motel (3.6%).

#### Incident Characteristics

Emergency medical services responded to the scene for a majority (71.4%) of all deaths of undetermined intent (Supplementary Table 10). Approximately half of these deaths occurred at the decedent’s home (49.7%); 14.7% of decedents were suspected of alcohol use preceding the incident, 7.2% were recently released from an institutional setting, and 5.5% had a child present or witnessing the incident. A small proportion of deaths of undetermined intent involved decedents experiencing homelessness (5.1%) or housing instability (2.5%) at the time of death.

#### Toxicology Results of Decedent

Toxicology tests for BAC were conducted for 62.3% of decedents in deaths of undetermined intent (Supplementary Table 11). Among those with positive results for alcohol (i.e., ethanol [37.7%]), 46.9% had a BAC ≥0.08 g/dL. The proportion of decedents tested for a substance varied and among those tested, the positive results differed by substance: amphetamines (39.2% tested, of which 35.8% were positive), anticonvulsants (29.2% tested, of which 27.0% were positive), antidepressants (34.1% tested, of which 44.5% were positive), antipsychotics (26.8% tested, of which 19.4% were positive), barbiturates (30.1% tested, of which 2.3% were positive), benzodiazepines (38.2% tested, of which 31.5% were positive), cannabis (commonly referred to as marijuana; 34.9% tested, of which 31.4% were positive), cocaine (46.5% tested, of which 38.8% were positive), muscle relaxants (25.5% tested, of which 9.3% were positive), and opioids (including illicit and prescription; 63.7% tested, of which 71.1% were positive). Carbon monoxide was tested for a substantially smaller proportion of decedents (5.3%) but was identified in 59.5% of those decedents. Results for other drugs for which <25% of decedents were tested are available (Supplementary Table 11).

#### Precipitating Circumstances

Precipitating circumstances were identified in 75.0% of deaths of undetermined intent (Supplementary Table 12). Among deaths of undetermined intent with known circumstances, 36.4% of decedents had a current diagnosed mental health problem at time of death. Among those with a diagnosed mental health problem, the most common diagnoses were depression or dysthymia (53.3%), anxiety disorder (27.5%), and bipolar disorder (24.0%); 7.3% had depressed mood at the time of death. Substance use problems (other than alcohol [63.3%]) and alcohol problems (24.4%) were commonly reported circumstances. Among all deaths of undetermined intent, 20.2% of decedents were receiving mental health or substance use treatment at the time of death, and 26.8% had a history of ever being treated for a mental health or substance use problem. The victim being known to authorities (16.8%) and a recent or impending crisis during the preceding or upcoming 2 weeks (12.8%) (Supplementary Table 13) were other life stressor circumstances identified in deaths of undetermined intent. Among decedents, 13.3% had a history of suicidal thoughts or plans, 8.4% had a history of attempting suicide, and 6.0% had disclosed intent to die by suicide.

Circumstances were identified in 211 (56.3%) undetermined deaths of children aged <18 years (Supplementary Table 12). Among child decedents, males had a higher percentage of substance use problems in their household compared with females (29.8% and 11.3%, respectively). Previous Child Protective Services involvement was more frequently reported in male decedents’ households than in female decedents’ households (18.3% and 11.3%, respectively).

### Homicides and Suicides in Puerto Rico

For 2022, Puerto Rico collected data on 727 incidents involving 809 deaths (data not shown). Homicide (n = 598) accounted for the largest proportion (73.9%) and highest rate (18.6 per 100,000 population) of these deaths, followed by suicide (n = 190 [23.5%; 5.9]) (Supplementary Tables 14 and 15).

#### Homicides 

##### Sex, Age Group, and Race and Ethnicity

In 2022, a total of 545 homicides among males and 53 homicides among females were reported in Puerto Rico (Supplementary Table 14). The overall homicide rate for males (35.8 per 100,000 population) was 11.5 times the rate for females (3.1). Among males, the homicide rate was 85.4 among those aged 18–29 years and 77.3 among those aged 30–44 years. Most (97.3%) homicide victims were Hispanic.

##### Method and Location of Injury

A firearm was used in a majority (93.6%) of homicides (Supplementary Table 14). A firearm was the most common method used in homicides of both males (94.3%) and females (86.8%); however, the firearm homicide rate for males (33.8 per 100,000 population) was 12.5 times the rate for females (2.7). Among males, a street or highway was the most common location of homicides (40.9%), but a motor vehicle or house or apartment was the most common location of homicides for females (both 34.0%).

##### Incident Characteristics

Emergency medical services responded to the scene for fewer than one fifth of male homicide victims (17.8%). A larger proportion of homicides among females than males occurred at the victim’s home (28.3% versus 8.8%, respectively). A small proportion of all homicide victims (all male) were in public custody when the injury occurred (2.3%) or were experiencing homelessness (2.2%).

##### Victim-Suspect Relationship

The victim-suspect relationship was known for 12.2% of homicides (Supplementary Table 14). When the relationship was known, the suspect for male victims was most often a person known to the victim, but the exact nature of the relationship was unclear (36.8%), followed by a stranger or other non–intimate partner relationship (both 26.3%). Among females, the suspect was most often a current or former intimate partner (75.0%).

##### Toxicology Results of Decedent

Tests for BAC were conducted for 84.3% of homicide decedents (Supplementary Table 16). Among those with positive results for alcohol (i.e., ethanol; 34.1%), 51.2% had a BAC ≥0.08 g/dL. The proportion of decedents tested for a substance varied and among those tested, the positive results differed by substance: cannabis (commonly referred to as marijuana; 86.0% tested, of which 43.4% were positive), cocaine (98.5% tested, of which 21.1% were positive), and opioids (98.3% tested, of which 8.2% were positive). Results for drugs for which <25% of decedents were tested are available (Supplementary Table 16).

##### Precipitating Circumstances

Precipitating circumstances were identified in 91.0% of homicides (Supplementary Table 17). Nearly half (47.1%) of male victims were known to authorities. Among males, homicides also commonly involved drugs (45.3%), were gang related (38.2%), or were precipitated by a relationship problem with someone other than a family member or intimate partner (21.7%). Intimate partner violence was identified as a contributing factor in 6.6% of homicides overall; intimate partner violence precipitated 29.8% of homicides among females, compared with 4.4% of homicides among males.

#### Suicides

##### Sex, Age Group, and Race and Ethnicity

In 2022, a total of 190 suicides (162 suicides among males and 28 suicides among females) were reported in Puerto Rico (Supplementary Table 15). The suicide rate for males was 6.6 times the rate for females (10.6 versus 1.6 per 100,000 population). Suicide rates were highest among males aged ≥65 years (15.4), followed by males aged 30–44 years and 45–64 years (both 13.5). The majority (96.8%) of all suicide decedents were Hispanic.

##### Method and Location of Injury

Hanging, strangulation, or suffocation was the most commonly used method for suicide among both males (62.3%) and females (53.6%) (Supplementary Table 15). A firearm was used in 23.5% of suicides among males. The most common suicide location was a house or apartment for both males (79.6%) and females (78.6%).

##### Incident Characteristics

Suicide decedents were commonly injured at their homes (73.7%) (Supplementary Table 15). Emergency medical services were more commonly present at the incident for females than for males (39.3% versus 24.1%).

##### Toxicology Results of Decedent

Tests for BAC were conducted for 80.0% of suicide decedents (Supplementary Table 18). Among those with positive results for alcohol (i.e., ethanol; 27.6%), 50.0% had a BAC ≥0.08 g/dL. The proportion of decedents tested for a substance varied and among those tested, the positive results differed by substance: cannabis (commonly referred to as marijuana; 88.4% tested, of which 11.9% were positive), cocaine (96.8% tested, of which 10.9% were positive), and opioids (96.3% tested, of which <10 decedents had positive results). Results for drugs for which <25% of decedents were tested are available (Supplementary Table 18).

##### Precipitating Circumstances

Precipitating circumstances were identified in 89.5% of suicides (Supplementary Table 19). Among decedents with known circumstances, approximately half were experiencing a depressed mood (50.6%) or had a current diagnosed mental health problem (46.5%).

Among males, 47.9% of suicide decedents had a current depressed mood, and 42.4% had a current diagnosed mental health problem. Depression or dysthymia was most often the mental health diagnosis for male suicide decedents with a diagnosed mental health problem (77.0%), followed by anxiety disorder (21.3%). More than one fourth of male suicide decedents had a history of ever being treated for a mental health or substance use problem or of expressing suicidal thoughts and plans (both 29.2%). Approximately one fourth (25.7%) of male decedents had a history of attempting suicide. Other precipitating circumstances for male suicide decedents included current mental health or substance use treatment (22.9%) and alcohol problems (18.1%).

Among female suicide decedents, 69.2% had a current diagnosed mental health problem and 65.4% had a current depressed mood. Depression or dysthymia was most often the mental health diagnosis for female suicide decedents who had a diagnosed mental health problem (72.2%). Half (50.0%) of female decedents had a history of expressing suicidal thoughts or plans, 46.2% had a history of attempting suicide, and 38.5% had ever been treated for a mental health or substance use problem.

## Discussion

Suicides, homicides, legal intervention deaths, unintentional firearm deaths, and deaths of undetermined intent affect all persons, regardless of sex, age, educational level, or race and ethnicity. NVDRS provides information on specific manners of death and can be used to describe characteristics of specific populations particularly affected by these fatal injuries. NVDRS data also can identify common circumstances for these manners of death. These details increase the knowledge base about the circumstances associated with these deaths and can assist public health authorities and their partners in developing and informing effective, data-driven approaches to violence prevention.

The occurrence of deaths captured by NVDRS varies greatly across states, the District of Columbia, and Puerto Rico (*1*). This report summarizes data on deaths that occurred in 2022 in all 50 states, the District of Columbia, and Puerto Rico, and describes selected characteristics. The 47 states with statewide data; the counties covered in California, Florida, and Texas; and the District of Columbia represented 90.9% of the U.S. population (*10*) and accounted for 91.3% of violent deaths and suicides in the United States in 2022 (*1*). NVDRS contributes to measurement of the national prevention initiative Healthy People 2030 objectives related to reducing the rates of suicides, homicides, firearm-related deaths, and deaths related to child abuse and neglect (*14*).

These injury-related deaths are preventable, and reducing deaths in communities is possible with evidence-based approaches (*15*). CDC has developed Prevention Resources for Action to assist communities in identifying prevention approaches that are based on the best available evidence. These resources describe strategies and specific programs, practices, and policies with evidence to reduce the risk for suicide, community violence, child abuse and neglect, adverse childhood experiences, intimate partner violence, and sexual violence (*16*–*21*). Each Prevention Resource for Action considers the multifaceted and interactive effects of the different levels of social-ecological interrelationships, including individual, relationship, family, school, and community factors that influence violence-related outcomes. NVDRS gathers ongoing, systematic, and consistent data on deaths that can be used by prevention experts within their communities to inform planning and implementation and track outcomes of prevention strategies and approaches.

### Suicides

#### Suicide Circumstances

Approximately one third of suicide decedents had a history of suicidal thoughts or plans, and one fifth had disclosed their suicidal intent. Multiple factors contribute to the risk for suicide (*22*), and the findings in this report indicate that recent or impending crises, intimate partner problems, physical health problems, and arguments or conflicts were common precipitating circumstances. Although one of the most commonly identified circumstances was a currently diagnosed mental health problem, approximately half of suicide decedents were not known to have a diagnosed mental health problem at the time of death. Past suicidal behavior and mental health problems are well documented as important risk factors to emphasize in suicide prevention (*18*,*22*,*23*). Less than one fourth of suicide decedents were known to be receiving treatment at the time of death, indicating a gap between those receiving treatment and those who would likely benefit from it.

Mental health problems and substance use also often co-occur among suicide victims (*24*,*25*). In this analysis, alcohol use, especially alcohol use in excess of the legal limit (i.e., BAC ≥0.08 g/dL), was frequently observed among suicide decedents who were tested for substances. Although examination of antemortem versus postmortem BAC results was beyond the scope of this report, it is likely that a substantial proportion of the toxicology testing for suicide decedents in this analysis was performed postmortem (e.g., the decedent did not survive long enough to receive antemortem testing at a hospital). Other research examining antemortem and postmortem BACs among fatally injured persons has found that postmortem BACs underestimate alcohol involvement (*26*), which suggests that this report’s findings of BACs in excess of the legal limit might have been conservative. Alcohol use is a strong predictor of suicidal behavior (*27*,*28*). Alcohol use might cause disinhibition, increase negative feelings related to oneself or others, and lead to impulsive behaviors or might stem from risk factors that are also risk factors for suicide (e.g., depression and adverse childhood experiences) (*29*). In addition, positive toxicology results for opioids (illegal or prescription) were reported in nearly one fourth of suicide decedents tested for these substances. Previous research has suggested that chronic pain, which might lead to opioid use, might be a contributor to suicide (*30*). Important activities to address the opioid overdose epidemic include expanding naloxone availability and access to treatment with medications for opioid use disorder, enhancing public health and public safety partnerships, addressing prescription opioid misuse, and maximizing the ability of health systems to link persons who use drugs to treatment and harm-reduction services (*31*–*33*).

Another factor that contributes to the risk for suicide is access to lethal means (e.g., firearms) among persons at risk for suicide (*22*,*34*). A firearm was the most common method used in suicides, accounting for approximately half of the deaths by suicide included in this analysis, except in Puerto Rico where hanging, strangulation, or suffocation was the most common method. Because certain lethal means (e.g., firearms) have high case-fatality rates, they confer limited opportunity for immediate life-saving response or intervention (*34*,*35*). Males and older adults are more likely than females and younger adults, respectively, to use firearms as a means of suicide (*35*–*37*). This analysis found that suicide rates were highest among males and adults aged ≥75 years. Creating protective environments by reducing access to lethal means among persons at risk for suicide can be an effective strategy to prevent suicide (*34*,*38*).

Although this report focused largely on individual- and relationship-level characteristics, broader community- and society-level factors also influence suicide patterns and trends and could be considered for suicide prevention strategies. For example, a recent study on suicide and county-level factors found higher levels of health insurance coverage, broadband internet access, and household income were associated with lower suicide rates (*39*). 

#### Suicides of Young Children

This report is the first to present NVDRS suicide data for children aged <10 years. Emerging data indicate suicides occurring among young children represent a growing concern (*8*) and need further study regarding circumstances and risk factors (*40*,*41*). In 2023, the National Institute of Mental Health convened a 2-day workshop to discuss child and youth suicide, including suicides among children aged <10 years (*42*). In part because of recent studies providing evidence for the existence of suicidal thoughts and behaviors among preadolescents (*43*,*44*), workshop participants suggested that increased awareness is needed among death investigators, medical examiners, and coroners that suicides occur among children aged <10 years and a need exists for more thorough investigation and documentation of these deaths.

Suicide is a complex problem, and research is evolving regarding risk factors for young children. Certain studies have focused on mental health issues. Studies have reported that children (*45*–*49*), including preschool-age children (*50*–*55*), experience depression. Concerns exist that depression-related issues might continue (*54*) and put children at risk for suicidal thoughts and behaviors later in childhood (*43*,*44*,*56*). Childhood suicidal thoughts and behaviors put children at risk for adult suicidal thoughts and behavior (*57*,*58*). Although research suggests that certain young children might have the reasoning ability to understand the permanence of suicide (*56*), more research is needed to examine this issue. More research also is needed to examine other potential circumstances of suicide among children (e.g., electronic screen time), which has been reported as being associated with child-reported suicidality in children aged 9–10 years (*59*). However, the specific mechanism behind the findings remains unknown. Whether the findings could be attributed to increased exposure to cyberbullying, negative social comparisons, or factors such as social withdrawal or avoidance is unclear (*59*).

The topic of suicide among young children is one that must be broached with multiple caveats. As is the case with all death investigations, issues and barriers might make a designation of suicide challenging (e.g., difficulty gathering comprehensive investigative information). For instance, law enforcement and coroner and medical examiner reports are constrained by the information provided by key informants. However, these informants might not possess awareness of the challenges that the child decedent was experiencing. In addition, findings in this report indicate there were few suicides among children aged <10 years, limiting the ability to calculate rates and signaling that multiple years of data might be necessary to fully analyze these rare events. Nevertheless, examining the available data for suicides of young children presents an opportunity for understanding the circumstances surrounding these incidents and might help to inform, develop, and tailor prevention opportunities geared toward young children.

#### Racial, Ethnic, and Sex-Based Differences in Suicide Rates

Suicides comprised the majority of deaths collected in NVDRS and occurred at higher rates among males than among females and, in the states and the District of Columbia, among AI/AN, White, and NH/PI persons compared with persons of other racial and ethnic groups. Males have a heightened risk for suicide compared with females, which is influenced by diverse factors, including traditional norms of masculinity (e.g., the desire to be seen as in control) that drive reluctance to seek help for mental health problems (*60*). Specific risk factors contributing to higher rates of suicide among males include rurality, access to lethal means, alcohol and drug use, intimate partner relationship breakdown, depression, and lower education levels (*61*,*62*). Further research is needed to inform the development of tailored prevention strategies for males (*60*,*63*).

AI/AN persons had the highest rates of suicide overall and for each sex. These findings warrant attention to the contextual factors that might contribute to higher rates of suicide (e.g., barriers to accessing mental health care, exposure to the suicide of a friend or family member as a contributing factor to a person’s own death by suicide, relationship problems, and alcohol and substance use) (*64*). Among AI/AN persons, the intergenerational effects of trauma have contributed to hardships such as poverty, unemployment, and housing instability that likely increase risk for suicide (*64*–*68*). Effective prevention measures may need to not only take that collective trauma into account, but also tailor prevention efforts to the unique needs of the specific AI/AN population impacted (*65*,*69*).

NVDRS data on NH/PI decedents as a separate race and ethnicity category was reported for the first time in 2021 (*7*). However, the Hawaii VDRS data did not meet criteria for inclusion in the 2021 NVDRS report, which might have influenced the findings for NH/PI persons (*7*). For 2022, both the NH/PI category and data from the Hawaii VDRS are included for the first time, and findings are presented that provide greater support that NH/PI males have an elevated risk for suicide. Although NH/PI-specific suicide research has been limited because of common historical data aggregations of Asian or other racial and ethnic groups with NH/PI persons, disaggregated studies have found increased suicidal behavior among NH/PI persons (*70*,*71*). Certain risk factors experienced by NH/PI persons might be similar to those experienced by AI/AN persons (*67*).

#### Suicide Prevention Strategies

CDC’s Suicide Prevention Resource for Action: A Compilation of the Best Available Evidence identifies the following seven strategies for reducing suicide and suicidal behaviors: 1) strengthen economic supports, 2) create protective environments, 3) improve access and delivery of suicide care, 4) promote healthy connections, 5) teach coping and problem-solving skills, 6) identify and support persons at risk, and 7) lessen harms and prevent future risk (*18*). These strategies support the goals and objectives of the National Strategy for Suicide Prevention (NSSP), a comprehensive national agenda for suicide prevention, which was recently updated to incorporate new and expanded strategies, foci, and objectives and to strengthen and build on recent advances in suicide prevention, such as increased funding for research and prevention-related programming, expanded access to services (e.g., the National 988 Suicide and Crisis Lifeline), and data science innovations (e.g., use of near–real-time syndromic surveillance) (*23*).

The updated NSSP was accompanied by the first U.S. Federal Action Plan, which outlines hundreds of suicide prevention action steps for CDC and other Federal agencies to carry out over the next 3 years (*72*). NVDRS is relevant to NSSP’s goals of increasing timeliness and usefulness of surveillance systems related to suicide prevention and evaluating outcomes and effectiveness of suicide prevention interventions. CDC’s Suicide Prevention Resource for Action includes examples of specific approaches that communities can implement to use each strategy (*18*). The findings in this report underscore the importance of approaches outlined in the resource for action, such as programs to teach emotional regulation and coping skills, enhanced parenting skills and family relationships, treatment for persons at risk for suicide, and treatment to prevent reattempts.

CDC’s Comprehensive Suicide Prevention Program funds programs around the country to implement and evaluate a comprehensive public health approach to suicide prevention (*73*). The programs focus on populations disproportionately affected by suicide, such as veterans; tribal populations; rural communities; sexual minorities; and young adults, with the goal of reducing suicide and suicidal behaviors by 10% among these groups. The programs are encouraged to use NVDRS data to inform what factors contribute to suicide for each of these populations (*74*). Comprehensive suicide prevention includes building leadership to support partnerships, using data to identify and understand prevention-related needs, monitoring trends in suicides and suicidal behavior, implementing and evaluating the strategies outlined in the Suicide Prevention Resource for Action, and developing and evaluating communication and dissemination strategies (*73*). In 2024, CDC released guidance on the investigation and response to suicide clusters (*75*). Although not examined in this report, NVDRS was listed as a data source available for local assessment and investigation of suspected suicide clusters, further demonstrating the potential of NVDRS for supporting evidence-based suicide prevention (*76*,*77*).

### Homicides

#### Homicides of Infants and Children

Although homicide rates for children varied across age groups, infants (i.e., aged <1 year) experienced a higher homicide rate compared with children aged 1–14 years. Certain studies have found the highest risk for newborn and infant homicide is on the day of birth (*78*,*79*). Risk starts in infancy and continues throughout childhood, highlighting the need to prioritize strategies focused on the prevention and intervention of child abuse and neglect to reduce risk for morbidity and mortality (*19*). Child abuse and neglect often are associated with immediate physical injuries, emotional and psychological problems, involvement in risky health behaviors later in life, and a wide range of broader physical health challenges and long-term health consequences (*19*).

CDC’s Child Abuse and Neglect Prevention Resource for Action: A Compilation of the Best Available Evidence identified the following evidence-based strategies and approaches: 1) strengthening economic supports for families, 2) changing social norms to support parents and positive parenting, 3) providing quality care and education early in life, 4) enhancing parenting skills to promote healthy child development, and 5) intervening to decrease harms and prevent future risk (*19*). Child abuse and neglect are preventable, and the specific approaches described in the compilation can help create safe, stable, and nurturing relationships and environments to prevent physical, mental, and emotional injuries as well as homicides of infants and children. The lack of safe, stable, and nurturing relationships and environments, which are essential for promoting children’s health and well-being, puts children at risk for adverse childhood experiences including violence, abuse, or death.

CDC’s Adverse Childhood Experiences Prevention Resource for Action: A Compilation of the Best Available Evidence is a comprehensive approach to preventing and mitigating the harms of adverse childhood experiences (*17*). The immediate and long-term harms of adverse childhood experiences can be lessened using multiple strategies, such as strengthening economic supports for families through work policies; promoting social norms that protect against violence and adversity via public education campaigns; ensuring a strong start for children through programs (e.g., early childhood home visitation); providing access to quality and affordable child care and preschool enrichment programs; connecting youths to caring adults and activities; and intervening with enhanced primary care or victim-centered services (*17*).

#### Racial and Ethnic Differences in Homicide Rates

Racial and ethnic minority groups experience higher rates of violent injury and homicide, particularly among youths and young adult males (*80*). In the United States among both males and females, Black and AI/AN persons experienced the highest rates of homicide. The homicide rate for Black males was 2.5–29.5 times the homicide rate for males from other racial and ethnic groups. In Puerto Rico, the homicide rate was more than triple the suicide rate, and male victims, who were predominantly Hispanic, experienced homicide rates more than triple the homicide rates experienced by Hispanic males in the states and the District of Columbia. Racial and ethnic differences in exposure to violence are pervasive and persistent, and reducing this exposure is an important part of a comprehensive approach to preventing violence (*80*,*81*). 

A disproportionate number of racial and ethnic minority youths live in communities with concentrated poverty, stressed economies, residential instability, neighborhood disorganization, low community cohesion, and informal controls (*81*,*82*,*83*). All these conditions are associated with violence and violence-related injuries, and addressing these conditions can have broad and sustained effects in reducing racial and ethnic differences in violence exposure (*3*,*21*,*81*,*82*,*83*). Research shows that policies and programs that strengthen economic and household stability and improve physical and social environments can reduce the continuation of violence and these differences (*21*,*81*). In conjunction with other data sources, NVDRS data can be used to help states and jurisdictions identify and address salient circumstances related to violence at the neighborhood and community levels, which can contribute to greater population-level decreases in violence (*82*).

#### Intimate Partner Violence–Related Homicides

Homicides among males were most often precipitated by an argument or conflict or occurred during the enactment of a crime (predominantly assault or homicide), or, in Puerto Rico, by the victim being known to authorities or drug involvement. In contrast, the most commonly identified precipitating circumstance for female homicide victims was intimate partner violence, and a current or former spouse or intimate partner was the identified suspect in at least half of female homicides with known suspects. Estimates from the 2016/2017 National Intimate Partner and Sexual Violence Survey indicated that approximately 111 million persons in the United States have experienced intimate partner violence (e.g., contact sexual violence, physical violence, or stalking victimization by an intimate partner); furthermore, approximately 41% of females and 26% of males in the United States have experienced intimate partner violence and associated adverse effects, including the experience of fear or concern for safety, at some point in their lives (*84*). Moreover, other lives are endangered by intimate partner violence beyond those directly involved in the intimate partner relationship. For example, research has found corollary victims, such as children of those in the intimate partner relationship, might be victims of homicide related to intimate partner violence (*85*). Research suggests links to prior intimate partner violence and intimate partner homicides, including that greater accessibility of firearms for persons who perpetrate intimate partner violence (*86*,*87*) and stalking or threats by an intimate partner (*88*) might be associated with increased risk of intimate partner homicide. Thus, the prevention of nonfatal forms of intimate partner violence might potentially reduce intimate partner homicides.

CDC’s Intimate Partner Violence Prevention Resource for Action: A Compilation of the Best Available Evidence outlines multiple strategies for programs and policies to prevent intimate partner violence and to decrease harms (*20*). Strategies and approaches to prevent and reduce intimate partner violence might occur across different levels of social-ecological interrelationships, such as engaging men and boys as allies (*20*,*89*); disrupting developmental pathways toward intimate partner violence; creating protective school, workplace, and neighborhood environments (*20*); teaching youth about safe and healthy relationships (*20*,*90*); empowering bystanders; and strengthening economic supports for families (*20*). 

#### Community Violence–Related Homicide Prevention Strategies

CDC’s Community Violence Prevention Resource for Action: A Compilation of the Best Available Evidence for Youth and Young Adults identifies the following seven strategies for preventing community violence and its impacts when violence has occurred: 1) strengthen economic security, 2) provide quality education, 3) create protective environments, 4) promote healthy family relationships, 5) strengthen youths’ and young adults’ skills, 6) connect young persons to caring adults and activities, and 7) intervene to lessen harms and prevent future risk. These strategies have been shown to prevent community violence or affect conditions or behaviors that increase risk for or protect against violence (*21*). This prevention resource describes the importance of a comprehensive approach that addresses societal factors (e.g., economic security and quality education) as well as strategies to reduce harms that can have short and long-term impacts (*91*). NVDRS is relevant to community violence prevention because the system collects information on community violence that describes and characterizes these incidents in a way that can help focus prevention activities where they are most needed.

The Community Violence Prevention Resource for Action includes examples of specific approaches that communities can implement within each strategy. The findings in this report underscore the importance of approaches outlined in the prevention resource for action, including providing tax credits to enhance family financial security or incentivize developers to provide affordable housing, increasing educational attainment for youths and young adults, reducing exposure to harmful community conditions, supporting school-based skill-building programs, and providing access to treatment to lessen the harms of violence for those who have been affected (*21*).

### Other Manners of Death

#### Legal Intervention Deaths

NVDRS collects more complete information on legal intervention deaths than other existing data sources in the United States (*92*). The rate of legal intervention deaths was highest for AI/AN persons, followed by Black persons. The rates for AI/AN males and Black males were 6.4 times and 2.8 times that for their White male counterparts, respectively, a finding consistent with previous studies (*93*,*94*). Racial and ethnic differences in fatal police shootings have been examined (*95*–*99*), and a systematic review of use of deadly force by law enforcement found that these differences might be associated with societal, community, and individual-level factors (*100*). NVDRS can help provide information on the context and circumstances of these deaths that can be used to help develop appropriate prevention strategies and monitor their effectiveness.

#### Unintentional Firearm Injury Deaths

Studies using NVDRS data have expanded understanding of unintentional firearm injury deaths, including details about victims and shooters. In this report, nearly half of unintentional firearm injury deaths were self-inflicted; however, approximately one third were inflicted by another person. Most of these deaths occurred while playing with the firearm, unintentionally pulling the trigger of the firearm, thinking the firearm was unloaded, or showing the firearm to others. These are concerning circumstances, particularly when children are involved as the shooter, victim, or both. These findings highlight the importance of secure storage practices and education about safe handling of firearms (*101*,*102*).

#### Deaths of Undetermined Intent

Poisoning was the method of injury in over half of deaths of undetermined intent in this report. Among those tested for each substance, a notable proportion of decedents had positive results for antidepressants, cocaine, or opioids (illegal or prescription) at the time of death. Research has demonstrated the challenges and variations in the classification of undetermined intent, particularly those due to poisoning, by U.S. coroners and medical examiners (*103*,*104*). These variations can be influenced by the definition of deaths of undetermined intent; the impact of decentralization in medicolegal practices; the differences in death investigation practices, training, and philosophies; and the subjectivity in categorizing deaths (*104*,*105*). The complexity of classifying deaths of undetermined intent is an important consideration for prevention efforts.

## Limitations

The findings in this report are subject to at least eight limitations. First, California, Florida, and Texas captured data from a subset of counties and those counties are not representative of all deaths occurring in these states. Therefore, the data in this report are not nationally representative.

Second, the availability, completeness, and timeliness of data depend on partnerships among VDRS programs and local health departments, vital statistics registrars’ offices, coroners and medical examiners, and law enforcement personnel. Data sharing and communication among partners are particularly challenging when states and U.S. territories have independent (instead of centralized) county coroner or medical examiner systems, numerous law enforcement jurisdictions, or both. NVDRS incident data might be limited or incomplete for areas in which these data-sharing relations are not fully developed. Partnerships with local vital statistics registrars’ offices usually are more established because they are part of the public health infrastructure. As part of an active surveillance system, VDRS programs work closely with local vital registrars’ offices to identify deaths that meet the NVDRS case definition and to avoid cases being missed or inappropriately included. CDC also monitors case ascertainment and variable completeness through regular technical assistance calls, which include reviews of the internal data quality dashboard in the web-based system that is updated in real time. Overall, core variables that represent demographic characteristics (e.g., age, sex, and race and ethnicity) and manner of death were known for >99% of cases.

Third, toxicology data are not collected consistently across all states, the District of Columbia, and Puerto Rico or for all alcohol and drug categories. In addition, toxicology testing is not conducted for all decedents nor is toxicology testing performed consistently among decedents who received testing (e.g., differences might exist in toxicology methodology used, levels of detection, and substances or metabolites tested). Thus, percentages of decedents with positive results for specific substances might be affected by resources available in the state or jurisdiction, infrastructure barriers, and testing practices in coroner or medical examiner offices (*104*). Moreover, NVDRS does not collect quantitative levels of the substances (other than alcohol) found to be positive. Therefore, this analysis could not determine whether decedents with positive medication-related toxicology were using prescriptions as directed or whether the positive result reflected alternative uses.

Fourth, abstractors are limited to the data included in the investigative reports they receive. In addition, reports might not fully reflect all information known about an incident, particularly for homicides and legal intervention deaths, when data are less readily available until a full investigation and adjudication are completed. Moreover, all VDRS programs in this report met the inclusion criteria of having at least 50% of cases with circumstance information collected from the coroner or medical examiner report or law enforcement report, and many VDRS programs exceeded that threshold. Still, the lack of complete circumstance data for all cases limits the representativeness of the circumstance data.

Fifth, case definitions present challenges when a single death is classified differently in different documents (e.g., unintentional firearm injury death in a law enforcement report, homicide in a coroner or medical examiner record, and undetermined on the death certificate). NVDRS abstractors reconcile these discrepancies using standard NVDRS case definitions and select a single manner of death based on all source documents (*9*).

Sixth, variations in coding occur depending on the data abstractor’s level of experience. For this reason, CDC provides extensive guidance and training, a coding manual to promote standardized data collection (*9*), and data validation checks. As part of their internal data quality efforts, VDRS programs are required to reabstract at least 5% of cases to examine consistency in coding and identify training needs of data abstractors.

Seventh, suicide deaths of young children (aged <10 years) were included in this analysis. Nine deaths were reported as suicides among children aged 5–9 years, so rates for this age group were suppressed. In addition, this change meant that suicide rates were computed using a population of all ages, which differed from previous NVDRS reports. Careful interpretation is advised when comparing these results with previous NVDRS reports and with other studies that do not have the same age criterion. The science regarding such deaths is an evolving area of study (*42*).

Finally, medical and mental health information (e.g., type of condition and whether the decedent was receiving treatment) often are not captured directly from medical records but from coroner or medical examiner records and the decedent’s family members and friends. Therefore, the completeness and accuracy of this information are limited to the knowledge of the informant.

## Using VDRS Data for Action

States and jurisdictions have used VDRS data to generate reports and data visualizations to examine deaths and develop prevention strategies. For example, North Carolina VDRS produced numerous fact sheets highlighting data related to populations disproportionately affected by violence and making death data accessible (*106*). Examples of topics addressed include firearm injury deaths, suicides in urban and rural communities, older adult suicides, veteran suicides, youth homicides, intimate partner homicides, deaths by race and ethnicity, and deaths by county. Montana VDRS developed a dashboard that reports death rates in the state by county, year, and manner of death (*107*). This dashboard also captures data from eight AI/AN reservations and has been instrumental in highlighting that counties that house tribal nations are among those with the highest suicide rates.

VDRS program dashboards and reports can facilitate innovative methods to bring attention to prevention. For example, Arizona VDRS collaborated with Arizona State University’s Walter Cronkite School of Journalism and Mass Communication to produce the Public Broadcasting Service documentary “Life Is: Confronting Teen Suicide in Arizona” (*108*). Arizona VDRS statewide suicide data were instrumental in illustrating the need for the documentary. For example, data were used to highlight that the rate of suicide among adolescents in Arizona is consistently and significantly higher than the national average. Other VDRS programs have also leveraged their data for public-facing prevention campaigns. Vermont VDRS data were used in a suicide data linkage report, which found that 41% of Vermonters who died by suicide had reported suicidal ideation to a family member or loved one, and 29% told someone they were planning to take their life within a month of death (*109*). The analysis of this data contributed to developing the Facing Suicide VT — Give Help Campaign (*110*). This initiative focuses on educating friends and families about warning signs of suicidal ideation, providing practical tools to support persons struggling with thoughts of suicide, and connecting them with resources.

VDRS programs have taken leadership roles within their states to facilitate collaborations and to inform policies to prevent violence. For example, to commemorate North Carolina VDRS’s 20th-year milestone, the state VDRS hosted the conference event “The Future of Violence Prevention in NC: The Next 20 Years” (*111*). The conference convened local and national violence prevention partners, researchers, and practitioners to discuss current and future work related to firearm violence and injury prevention and safety in North Carolina. North Carolina VDRS also leveraged the practical opportunity to distribute 1,000 gun locks during this event. 

## Future Directions

Surveillance systems must be flexible to evolve according to changing needs of society and the public health community (*112*). As a web-based system, NVDRS continues to innovate internally and externally and will continue to participate in data modernization activities. For the 2022 data year process, CDC conducted selected data quality assurance and cleaning processes through the use of informatics tools resulting in increased efficiency and decreased data processing time. Natural language processing is being explored as a tool for analyzing incident narratives. During the past few years, certain VDRS programs have developed public-facing data dashboards that provide a snapshot of their jurisdiction’s VDRS data. These dashboards are used to inform public health partners, violence prevention practitioners, and the general public about violent deaths and suicides in their state (some present data at the city level). The ultimate goal of NVDRS is to use data for public health action.

Finally, this report summarizes data on violent deaths and suicides covered by NVDRS that occurred in 2022. This is the first year that data from all 50 states, the District of Columbia, and Puerto Rico met the NVDRS inclusion criteria. However, data from California, Florida, and Texas are only from a subset of counties and are not statewide. The goal is to include data for all counties from participating states in future reports to achieve full national representation.

## Conclusion

Public health surveillance is the foundation for public health practice (*113*). Monitoring the prevalence of violence-related fatal injuries, defining priorities, and informing prevention activities are essential parts of public health surveillance. In 2018, NVDRS received funding for nationwide expansion beginning with data collection in 2019. However, not all VDRS programs’ data met the inclusion criteria for reporting during data years 2019–2021. In 2022, for the first time, data from all 50 states, the District of Columbia, and Puerto Rico met the inclusion criteria and were included in this surveillance report. This expansion makes death information available for local communities to develop prevention efforts and allows for the system’s capacity to measure the need for and effects of violence prevention policies, programs, and practices at the national level.

## Footnotes

* Frequencies and rates of deaths included in this report differ slightly from the frequencies and rates of deaths reported by CDC’s WISQARS, which excludes non-resident deaths (i.e., occurrent deaths) that occur in participating states and the District of Columbia. NVDRS tracks both resident and occurrent deaths in the overall data set, and the numbers in this report reflect both. VDRS programs are expected to collect information on deaths among their residents and fatal injuries occurring within their borders irrespective of the decedent’s residence status. The state of injury occurrence is responsible for collecting the information. By making this differentiation of responsibility, duplicate reporting in NVDRS is avoided.

† The name of the Institute of Medicine was changed to the National Academy of Medicine, effective July 1, 2015.

§ To be included in NVDRS, deaths of undetermined intent must have evidence of the possibility that the intent was purposeful, including use of a weapon or other evidence that force was used to inflict the injury. Most commonly, the coroner or medical examiner is unsure whether the death was a suicide or unintentional.

¶ 45 C.F.R. part 46.102(l)(2), 21 C.F.R. part 56; 42 U.S.C. Sect. 241(d); 5 U.S.C. Sect. 552a; 44 U.S.C. Sect. 3501 et seq.

** Drug trade is defined as the buying, selling, or passing of drugs from one person to another in exchange for goods or money.
